# Pumilacidins from the Octocoral-Associated *Bacillus* sp. DT001 Display Anti-Proliferative Effects in *Plasmodium falciparum*

**DOI:** 10.3390/molecules23092179

**Published:** 2018-08-29

**Authors:** Daniel Torres-Mendoza, Lorena M. Coronado, Laura M. Pineda, Héctor M. Guzmán, Pieter C. Dorrestein, Carmenza Spadafora, Marcelino Gutiérrez

**Affiliations:** 1 Centro de Biodiversidad y Descubrimiento de Drogas, Instituto de Investigaciones Científicas y Servicios de Alta Tecnología (INDICASAT AIP), Panama, Apartado 0843-01103, Panama; dtorres@indicasat.org.pa; 2 Department of Biotechnology, Acharya Nagarjuna University, Nagarjuna Nagar, Guntur 522510, India; 3 Centro de Biología Celular y Molecular de Enfermedades, INDICASAT AIP, Panama, Apartado 0843-01103, Panama; lcoronado@indicasat.org.pa (L.M.C.); lpineda@indicasat.org.pa (L.M.P.); 4 Smithsonian Tropical Research Institute, Balboa, Ancon P.O. Box 0843-03092, Panama; guzmanh@si.edu; 5 Collaborative Mass Spectrometry Innovation Center, Skaggs School of Pharmacy and Pharmaceutical Sciences, University of California at San Diego, La Jolla, CA 92093, USA; pdorrestein@ucsd.edu; 6 Department of Pharmacology, University of California at San Diego, La Jolla, CA 92093, USA

**Keywords:** *Bacillus*, surfactins, pumilacidins, coral-associated bacteria, *P. falciparum*, apoptosis

## Abstract

Chemical examination of the octocoral-associated *Bacillus* species (sp.) DT001 led to the isolation of pumilacidins A (**1**) and C (**2**). We investigated the effect of these compounds on the viability of *Plasmodium falciparum* and the mechanism of pumilacidin-induced death. The use of inhibitors of protein kinase C (PKC) and phosphoinositide 3-kinase (PI3K) was able to prevent the effects of pumilacidins A and C. The results indicated also that pumilacidins inhibit parasite growth via mitochondrial dysfunction and decreased cytosolic Ca^2+^.

## 1. Introduction

Surfactins are cyclic lipopeptides that consist of a heptapeptide backbone and one β-hydroxyl fatty acid with variable chain lengths (12–16 carbons) produced by members of the *Bacillus* genus [1,2,3,4,5]. These compounds display a broad spectrum of biological activities, such as antibacterial [6,7], antifungal [8,9,10], antiviral and anti-ulcer [11], hemolytic [12], antitumor [13], anti-mycoplasma [14], anti-inflammatory [15,16,17,18], and insecticidal [19], and they have neuroprotective effects [20] among other properties that make them promising for industry, biotechnology, and pharmacy [21]. Pumilacidins are a variant of the surfactin family that consist of a β-hydroxy fatty acid, l-glutamic acid, l-leucine, d-leucine, l-leucine, l-aspartic acid, d-leucine, and l-isoleucine (or l-valine), and they exhibit antiviral activity against herpes simplex virus type 1 and inhibitory activity against hog gastric H^+^, K^+^-ATPase [11], and recently, antibacterial activity against *Staphylococcus aureus* [22].

In living organisms, apoptosis is defined as a caspase-dependent variant of regulated cell death, triggered by intrinsic or extrinsic events. Apoptosis is highly controlled, but it is also reversible, as demonstrated by the existence of metabolic checkpoints that function to reestablish homeostasis [23]. This apoptosis-like process was described for some parasites, such as *Trypanosoma* and *Leishmania*, as well as *Plasmodium* [24,25,26,27].

Intracellular signals regulating cell survival and death are now largely understood, owing to the numerous published studies on the matter [28,29,30]. Of the signaling molecules, phosphoinositide 3-kinase (PI3K) is an important component in regulating the expression level and activation of apoptosis. In a previous study, it was found that a surfactin analog isolated from *Bacillus licheniformis* BC98 was a novel *Plasmodium falciparum* silent information regulator 2 (PfSir2) inhibitor [31]. Sir2-like proteins are histone acetyltransferases and deacetylases that catalyze the oxidized nicotinamide adenine dinucleotide (NAD^+^)-dependent deacetylation of lysine residues in target proteins, and they have a wide range of functions in cell activities including antigenic variation, gene silencing, cell-cycle progression, chromosome segregation, microtubule organization, protein-aggregate transport, genome stability, DNA repair, apoptosis, and autophagy. They are also related to aging, life-span, and metabolism studies. Moreover, some diseases like obesity, type II diabetes, cancer, and neurodegenerative disorders are linked to them [32,33,34]. Even with so many possible functions in *P. falciparum*, the deletion of the *PfSir2* gene is not lethal to the parasite [31,35], thus opening up the possibility of the presence of a similar gene in the *Plasmodium* genome, one not yet biochemically characterized and which would be the target of the surfactins. Since surfactins are implicated in inducing apoptosis and cell-cycle arrest in other cell types, their mechanism of action in *Plasmodium falciparum* parasites is still not clear [36]. In this study, we aimed to explore the anti-proliferative activity of the surfactins pumilacidin A (**1**) and pumilacidin C (**2**) against the malaria parasite, *Plasmodium falciparum*, and its mechanism of growth inhibition at the molecular level.

## 2. Results

### 2.1. Collection and Identification of Biological Material

The strain DT001 was isolated from the octocoral *Muricea fruticosa* (order Alcyonacea, family Plexauridae) collected in the surroundings of the submarine mountain Hannibal Bank in the Coiba National Park, Veraguas, Panama, in March 2012. The bacterium was identified as *Bacillus* species (sp.) based on its 16S ribosomal RNA (rRNA) gene sequence analysis.

### 2.2. Extraction, Isolation, and Characterization of Compounds

The strain *Bacillus* sp. DT001 was cultured in 10 L of M1 culture broth for 10 days in an orbital shaker at 150 rpm at room temperature, and then extracted with ethyl acetate to obtain the organic crude extract. The extract was fractionated using C-18 solid-phase extraction (SPE) cartridges by eluting in a stepwise gradient of MeOH–H_2_O followed by reverse-phase HPLC purification to obtain pumilacidins A (**1**) and C (**2**) (Figure 1). The structures of compounds **1**–**2** were deduced by spectroscopic analyses including one- and two-dimensional (1D and 2D) NMR, tandem MS, and by comparing (Figures S1–S18) with literature data [11,16,37,38].

### 2.3. Pumilacidins Inhibit the Growth of Plasmodium falciparum Parasites without Affecting Uninfected Cells

**Figure 2:**
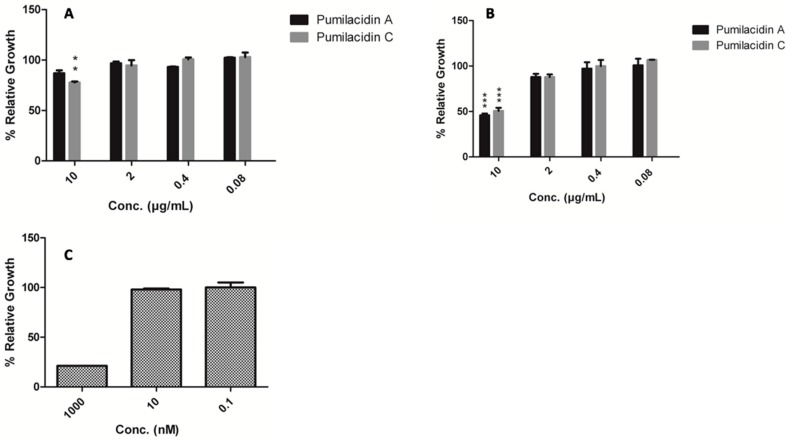
Effect of pumilacidins on the proliferation of *Plasmodium falciparum*. (**A**) Ring stage; (**B**) schizont stage; (**C**) chloroquine (CQ) control in ring stage. *Plasmodium* parasites were incubated together with pumilacidins A and C at 10, 2, 0.4, and 0.08 μM, and CQ at 1000, 10, and 0.1 nM for 48 h. The proliferation of pumilacidin-treated parasites was measured by PicoGreen DNA fluorometric measurement. Data represent the means ± standard error of the mean (SEM) of one representative observation out of three experiments performed in duplicate. ** *p* < 0.01, *** *p* < 0.001.

To assess the inhibitory effect of pumilacidins A and C on the growth of *Plasmodium falciparum*, parasites were treated with these compounds at different concentrations for 48 h, and a viability assay was conducted. The IC_50_ value obtained when the treatment was applied in the ring stage was 15.44 μM for pumilacidin A and 19.59 μM for pumilacidin C. For the schizont stage, the corresponding IC_50_ values were 8.34 μM for pumilacidin A and 7.75 μM for pumilacidin C (Table 1). These values are in accordance with previous work [31], and indicate that pumilacidins can suppress the proliferation of *Plasmodium falciparum* in a dose-dependent manner (Figure 2).

**Figure 3:**
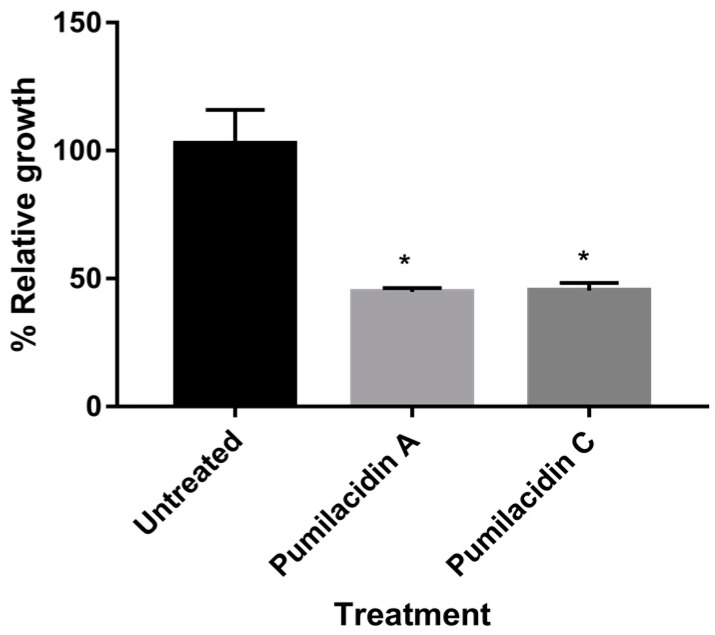
Effect of pumilacidins on the proliferation of *P. falciparum*. *Plasmodium* parasites were cultured in the presence of pumilacidins A and C at 10 μM for 24 h. The proliferation of pumilacidin-treated parasites was measured by flow cytometry quantifying their DNA content with Hoechst 33342 staining. Data represent the means ± SEM of two independent observations performed in triplicate. * *p* < 0.05.

For the mechanism-of-action studies, based on the screening results, we used 10 µM and schizont stage parasites to ensure pronounced effects that could be recorded, with the minimum amount of sample, incubating for only 24 h. As shown in Figure 3, compared with untreated cells, those treated with pumilacidins A and C at 10 μM for 24 h underwent almost 50% inhibition of parasite proliferation. 

**Figure 4:**
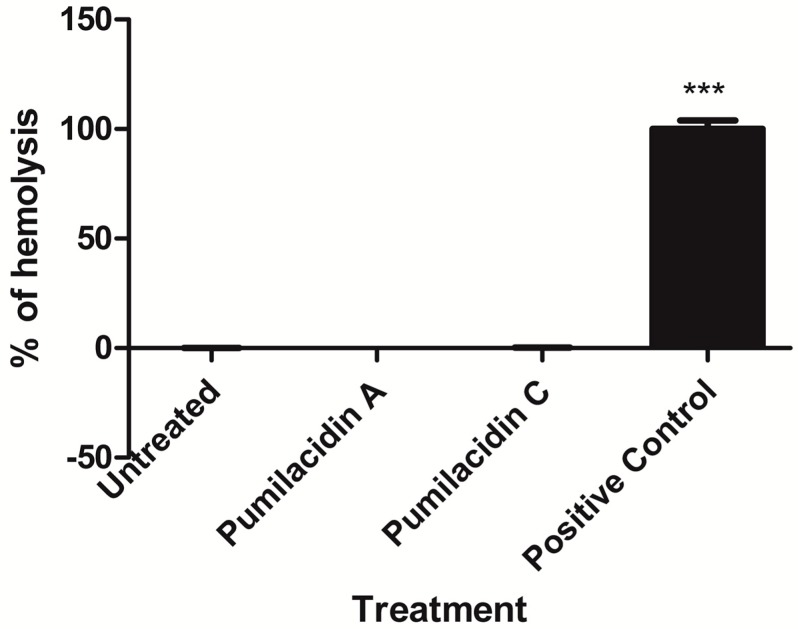
Effect of pumilacidins on hemolytic activity in red blood cells (RBCs). Red blood cells were incubated with pumilacidins at a concentration of 10 μM for 24 h. Data represent the percentage of hemolysis relative to 0.1% Triton as a positive control (100%) of one representative experiment run in triplicate. *** *p* < 0.001.

**Figure 5:**
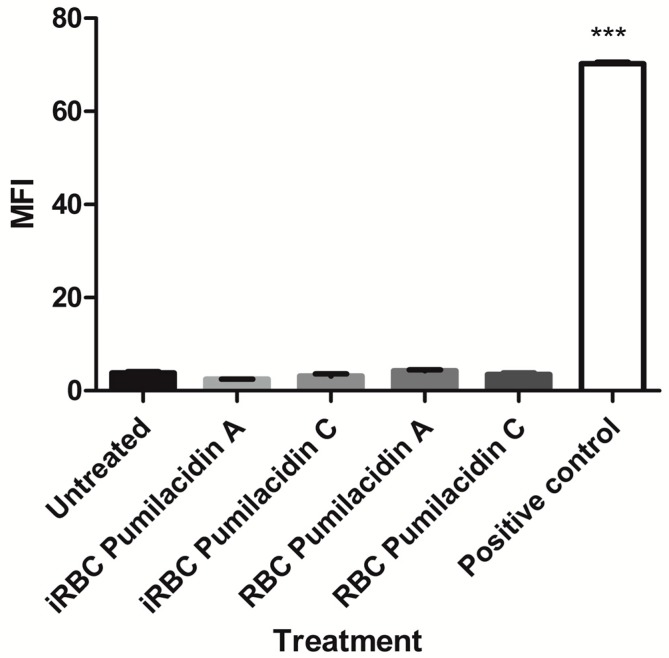
Effect of pumilacidins on cell-membrane integrity. Infected and uninfected red blood cells were cultured with pumilacidins A and C at 10 μM for 3 h. As a positive control, heating at 80 °C was used. Integrity of the membranes was determined by the incorporation of propidium iodide (PI) measured by flow cytometry and plotted as mean fluorescence intensity (MFI). Data represent the means ± SEM of one representative experiment performed in triplicate. *** *p* < 0.001.

As pumilacidins were tested in macrophages [16], we tested the effect of pumilacidins in uninfected red blood cells (uRBCs) and in another mammalian cell line, Vero cells. Our results showed that there was no evident negative effect against uRBCs. We did not find hemolytic activity against uninfected red blood cells when incubated with pumilacidins A and C at 10 μM (Figure 4). To assess any possible damage to RBCs in the form of pores in their membrane, the incorporation of propidium iodide (PI) was monitored. We did not find any significant change in the amount of PI in treated cells in comparison with the untreated control (Figure 5).

There was also no deleterious effect of pumilacidins A and C on normal epithelial cells. Based on our cytotoxic assay, the IC_50_ values for the compounds tested on these cells were 28.42 and 26.07 μM, respectively (Table 2).

### 2.4. Effect of Pumilacidins on the Induction of Pro-Apoptotic-Like Activity

**Figure 6:**
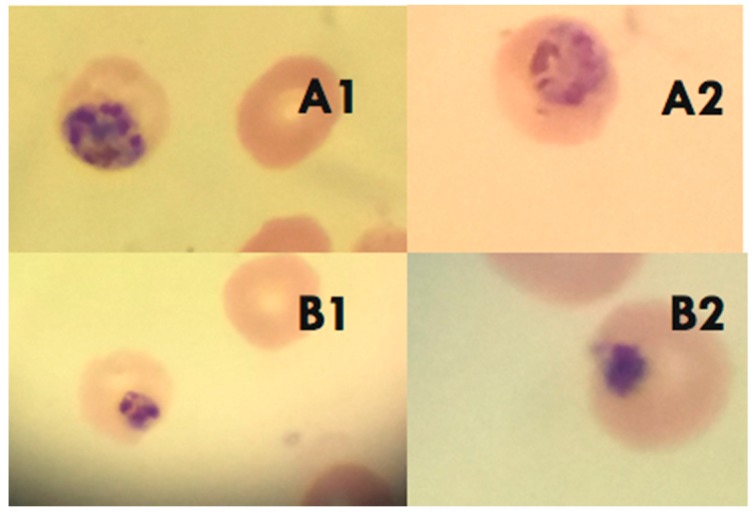
Morphological changes induced by pumilacidins A and C. Panel **A1** and **A2**: Untreated parasites. Panel **B1** and **B2**: *P. falciparum-*infected erythrocytes in the schizont stage were treated with 10 μM pumilacidins A and C, and their morphology was analyzed 48 h after treatment through Giemsa-stained smears.

To evaluate whether the pumilacidin-induced anti-proliferative effect was due to their triggering of proapoptotic-like activity, some apoptotic parameters such as morphological changes and alteration of membrane polarity were chosen for more in-depth analysis. Clear morphological changes (nuclear condensation and the presence of perinuclear apoptotic bodies) of *Plasmodium* parasites treated with pumilacidins at 10 μM were observed by conventional microscopy (Figure 6). The treatment was performed in the schizont stage, and morphological changes were analyzed 48 h later, in the schizont stage, where apoptotic signatures can be more easily distinguished.

### 2.5. Pumilacidin-Induced Apoptosis-Like Death through Mitochondrial Dysfunction Pathways

**Figure 7:**
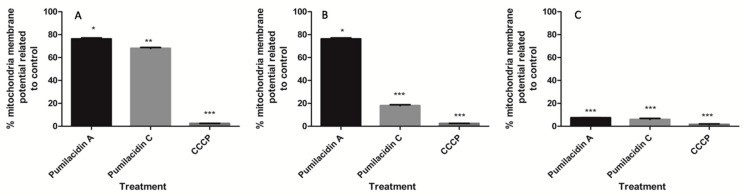
Mitochondrial membrane potentials. Changes in the mitochondrial membrane potentials relative to their untreated controls were measured with the mitochondrial potential-dependent dye DiO6 using flow cytometry after 3 h (**A**), 6 h (**B**), and 9 h (**C**) post treatment with pumilacidins A and C at 10 μM in the schizont stage of the parasites. * *p* < 0.05; ** *p* < 0.01; *** *p* < 0.001.

To determine whether pumilacidins induce the loss of mitochondrial membrane potential in *Plasmodium* parasites, we examined the effect of these surfactins on their potential using the Di06 dye, a fluorophore that accumulates rapidly and selectively inside the mitochondria depending on the membrane potential. We found that pumilacidin treatment caused disruption of the mitochondrial membrane potential as evidenced by a decrease in the proportion of cells with higher fluorescence intensity. This decrease occurred in a time-dependent manner showing a decreasing tendency from 3 h after treatment until reaching significance 9 h after treatment with surfactins (Figure 7).

Figure 7 shows the results expressed as percentage mitochondrial membrane potential compared to untreated controls, set as 100%. These values were calculated by subtracting the fluorescence of uRBCs from the infected RBCs (iRBCs) in each treatment and converting them to percentage relative to the controls.

### 2.6. Reactive Oxygen Species Generation Has No Role in the Effect of Pumilacidins on Parasites

**Figure 8:**
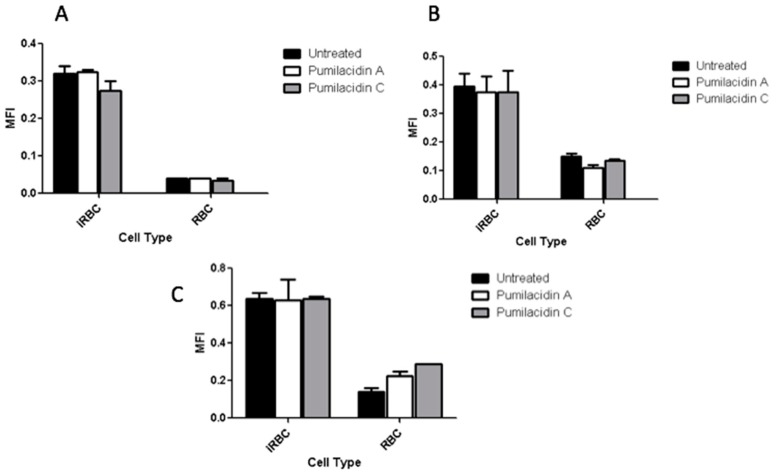
Reactive oxygen species (ROS) generation after surfactin treatment. After incubating *P. falciparum* parasites in the schizont stage with pumilacidins A and C at 10 μM for different time points, the levels of ROS were measured at 3 h (**A**), 6 h (**B**), and 9 h (**C**) post treatment with surfactins.

As shown in Figure 8, the treatment with pumilacidins induced no changes in the reactive oxygen species (ROS)-dependent dichloro-dihydro-fluorescein diacetate (DCFH-DA) fluorescence in a time-dependent manner. These results rule out the involvement of reactive oxygen species in the apoptosis-like death of malaria parasites after treatment with the surfactins.

### 2.7. Calcium Involvement in Parasite Death after Pumilacidin Treatment

**Figure 9:**
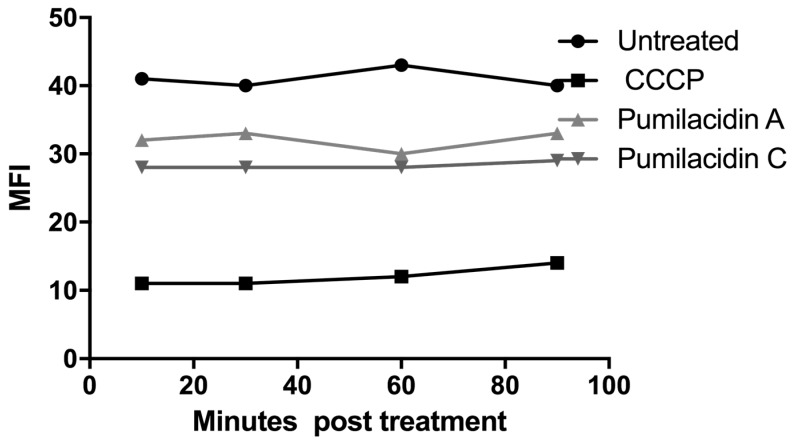
Kinetic measurement of cytosolic Ca^2+^. Saponin-isolated parasites stained with fura-2-acetoxymethyl ester (FURA 2AM) were treated with pumilacidins A and C at a concentration of 10 μM. Untreated parasites were used as controls. Readings were taken at 10, 30, 60, and 90 min after pumilacidin introduction. Samples were tested in duplicate. A representative result is shown.

To further determine the role of Ca^2+^ in pumilacidin-induced apoptosis, experiments were performed to see whether the surfactins could change Ca^2+^ concentration after treatment with pumilacidins A and C at 10 μM in saponin-isolated parasites. The levels of calcium measured through fluorescence intensity of the calcium dye fura-2-acetoxymethyl ester (FURA 2AM) were determined at 10, 30, 60, and 90 min after the introduction of surfactins. The results showed that, 10 min after the treatment with surfactins, there was a decrease in the amount of cytosolic calcium in the parasite for pumilacidins A and C when compared to the untreated controls, but with higher values than those obtained with the mitochondrial disruptor, carbonyl cyanide *m*-chlorophenyl hydrazone (CCCP) (Figure 9).

### 2.8. Effect of Pumilacidins on the Activation of Inositol Trisphosphate Apoptosis-Regulatory Signals

**Figure 10:**
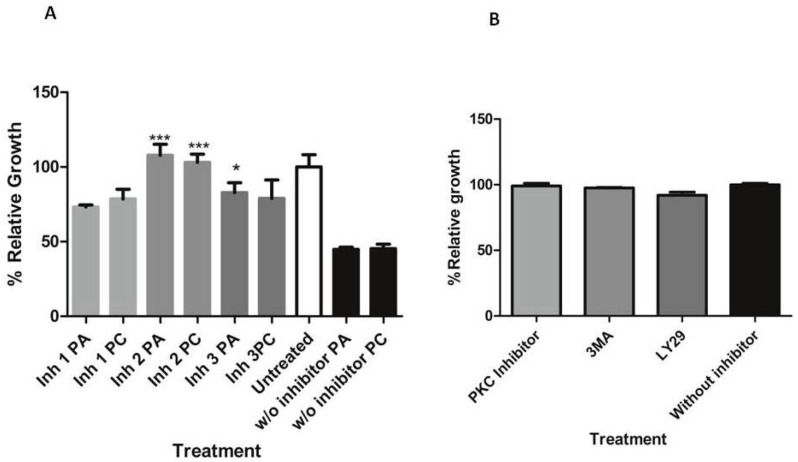
Erythrocytes infected with *P. falciparum* were either incubated without (w/o inhibitor) or pre-incubated with inositol triphosphate 3-kinase (IP3K; 5 μM) and protein kinase C (PKC; 4 μg/mL) inhibitors and then treated with pumilacidins A and C at 10 μM (**A**) or not (**B**). The growth of the parasites was analyzed by flow cytometric detection of DNA 24 h afterward, and compared to untreated culture controls incubated with each respective inhibitor. The bars represent the mean of three individual experiments in duplicate (**A**) or the mean of two individual experiments in duplicate (**B**). * *p* < 0.05; *** *p* < 0.001.

**Figure 11:**
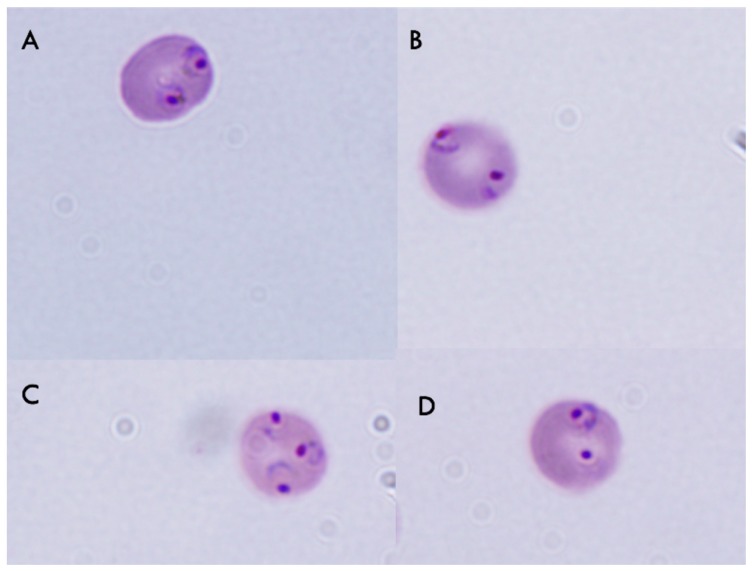
Morphology changes induced by treatment with PKC and phosphoinositide 3-kinase (PI3K) inhibitors. Erythrocytes infected with *P. falciparum* in the schizont stage were either incubated without (w/o inhibitor) or pre-incubated with (**A**) no inhibitor, (**B**) 3-methyladenine (3MA), (**C**) LY29400, and (**D**) bisindolylmaleimide I, and then treated with pumilacidins A and C at 10 μM for 24 h. The parasite morphology was analyzed by Giemsa smear under light microscopy.

Since it was reported that some signaling enzymes, such as the phosphatidylinositol 4,5-bisphosphate 3-kinase family, are known to play a central role in regulating pro-apoptotic processes including cell-cycle arrest, the modulatory effect of surfactins on the activation of these enzymes was examined by inhibiting this pathway and studying the effect of surfactins on parasite proliferation under this condition. To further assess the role of inositol triphosphate (IP3) enzymes in the surfactin-induced proliferation decrease, the inositol triphosphate 3-kinase (IP3K)-related inhibitors, 3-methyladenine and LY29400, and a protein kinase C (PKC) inhibitor were used since PKC has some reports of involvement with the IP3 apoptosis regulatory pathway [39] (See Table 3). The results described in Figure 10 show that all inhibitors tested partly reversed the surfactin-induced inhibition of growth of parasites after the treatment. The most significant reversal effect was obtained with the inhibitor, LY29400, that inhibits PI3-kinase class I. The inhibitors at the concentration used had no detrimental effect on parasites either in the normal proliferation or morphology, as shown in Figure 10B and Figure 11.

## 3. Discussion

Surfactins are reported to have several biological effects such as antifungal, antibacterial, anti-inflammatory, and antiparasitic activities (*Plasmodium*) [6,7,8,9,10,15,16,17,18]. In a study testing the anti-*Plasmodium* activity, the authors showed that surfactins from *Bacillus licheniformis* BC98 were able to inhibit the activity of the Sir2 enzyme [31]. Nonetheless, deletion of the PfSir2 gene is not lethal to the parasite [31,35], opening up the possibility of the presence of a second Sir2 gene present in the *Plasmodium* genome, not yet biochemically characterized, which would be the target of the surfactins. At this point, the mechanism of action of the surfactins in *Plasmodium* parasites is still not clear.

In this study, we showed that pumilacidins A and C isolated from *Bacillus* sp. DT001 inhibit the growth of malarial parasites in vitro. We found IC_50_ values for pumilacidins A and C (in the schizont stage) of 8.3 and 7.5 μM, respectively, which is comparable to the IC_50_ value obtained in a previous report [31]. Mitochondrial dysfunctions, including the loss of mitochondrial membrane potential, are associated with apoptosis [40]. To confirm the involvement of a mitochondrial signaling pathway during pumilacidin-induced apoptosis-like death, the reduction of its membrane potential was investigated. In this study, the loss of mitochondrial membrane potential was observed in the pumilacidin-treated cells.

We also found a decrease in cytosolic calcium from minute 10 after the treatment with pumilacidins A and C. This decrease might be due to calcium release from the endoplasmic reticulum (ER) or mitochondria, presumably through IP3 receptors. PI3Ks are a family of enzymes involved in cellular functions such as cell growth, proliferation, differentiation, motility, survival, and intracellular trafficking. PI3-kinase was immunolocalized, among other sites, within the food vacuole of the *Plasmodium* parasite [41]. PfPI3-kinase, a phosphatidylinositol 3-kinase in *P. falciparum,* was reported as a signaling protein involved in endocytosis and trafficking of hemoglobin. Inhibition of PfPI3-kinase resulted in attenuated hemoglobin digestion and the inhibition of parasite growth [41].

Considering that over 60% of all *P. falciparum* genes have no assigned function, it is not unusual that no canonical IP3 channel or receptor is identified at present in the *Plasmodium* genome, although pharmacological approaches suggested its presence [42]. Likewise, PKC isoforms are also critical regulators of cell proliferation and survival in other organisms, although no protein kinase C (PKC) is found in the *P. falciparum* genome database [43]. Nonetheless, another kinase may have taken its role, just as in plants, where it was suggested that another kinase, calcium-dependent protein kinase (CDPK), may function as PKC [44]. Since the growth rate of parasites is found to be decreased in PKC-deficient erythrocytes, it was also suggested that PKC from the red blood cell may be trafficked inside the erythrocyte, influencing the invasion ability of the *Plasmodium* parasite through the phosphorylation of the proteins ring-infected erythrocyte surface antigen precursor (RESA) and band 4.1, which increase the permeability of RBCs, making it possible for the parasite to invade [45].

The effect of surfactins could be explained on the basis of stimulated Ca^2+^ transport out of the cell through the plasma membrane and stimulated calcium reuptake into the ER or mitochondria. The release of calcium through IP3 receptor 1 is closely associated with the induction of apoptosis via the inner mitochondrial pathway. The activation of the mitochondrial pathway of apoptosis is deeply dependent on calcium release from the ER due to stress. The release of calcium from the ER and local interactions between the ER and mitochondria represent a key mechanism of apoptosis regulation. The PI3K inhibitors used were chosen because they are two of the most common PI3K inhibitors used in signaling studies. Moreover, based on our first mechanism results, we reasoned that the PI3K signaling pathway might be involved in the apoptotic-like death that we observed in surfactin-induced killing. The 3MA inhibitor has an inhibitory effect on class I and III phosphatidylinositol 3-kinases (PtdIns3Ks) [46] The LY294002 inhibitor is a potent and specific cell-permeable inhibitor of several isoforms of phosphatidylinositol 3-kinases (PI3-kinases) with IC_50_ values in the 1–50 μM range [47] which bind to the intracellular part of PI3K. LY294002 competitively inhibits ATP binding to the catalytic intracellular subunit of PI3-kinases and does not inhibit PI4-kinase, diacylglycerol (DAG)-kinase, PKC, protein kinase A (PKA), mitogen-activated protein kinase (MAPK), S6 kinase, epidermal growth factor receptor (EGFR), or cellular (c)-src tyrosine kinases and rabbit kidney ATPase. The inhibitor (bisindolylmaleimide I) used in this study targets all forms of PKC (α, βI, βII, and γ). Recently, the role of phosphorylation in PKC activation was examined, and it provided a clear link between PI3K/PtdIns-3,4,5-P3 signaling and PKC function [48]. Together, both inhibitors with their effect point to a role of PI3K in the chain of events that leads to apoptosis-like death. 

## 4. Materials and Methods

### 4.1. General Experimental Procedures

Optical rotations were measured in MeOH with a JASCO P-2000 digital polarimeter (JASCO, Easton, MD, USA). Ultraviolet (UV) spectra were recorded using a Shimadzu UV-Vis- spectrophotometer model UV-2401 (PC) (Shimadzu, Columbia, MD, USA). NMR spectra were recorded on an Eclipse 400 MHz spectrometer (JEOL, Peabody, MA, USA). Chemical shifts were calibrated internally based on the signal for the residual solvent in which the sample was dissolved (*d*-MeOH, δ_H_ 3.34, δ_C_ 49.86). Pre-fraction of the bacterial extract was carried out using SupelClean^TM^ C-18 solid-phase extraction cartridges (Supelco^®^ Analytical, Bellefonte, PA, USA). HPLC purification was carried out using an 1100 HPLC system (Agilent, Santa Clara, CA, USA) equipped with a quaternary pump, an Agilent 1200 Series diode array detector, and a reverse-phase silica gel column (Agilent Zorbax^®^ SB-C18, 4.6 mm × 150 mm). Tandem mass spectrometry (MS/MS) analysis of bacterial extracts and compounds was performed in a 6.42 T Thermo Finnigan LTQ-FT-ICR mass spectrometer (Thermo-Electron Corporation, San Jose, CA, USA) using a TriVersa NanoMate chip-based electrospray ionization source (Advion Biosystems, Ithaca, NY, USA) and an Agilent 1290 Infinity LC System (Agilent Technologies, Santa Clara, CA, USA) coupled to a micrOTOF-QIII mass spectrometer (Bruker Daltonics, Billerica, MA, USA) supplied with an electrospray ionization (ESI) source. Flow cytometry was performed in a CyFlow^®^ Space cytometer (Sysmex-Partec, Goerlitz, Germany).

### 4.2. Biological Material Collection and Identification

A healthy specimen of the octocoral *Muricea fruticosa* was collected by hand using scuba apparatus in the vicinity of the submarine mountain Hannibal Bank in the Coiba National Park on the Pacific coast of Veraguas, Panama in 2012. The coral specimen was identified as *Muricea fruticosa* (Verril 1869), based on its morphology. For the isolation of the coral-associated bacteria, we followed a protocol previously reported [49,50,51], using the nutrient medium M1 (10 g of potato starch, 4 g of yeast extract, 2 g of peptone from potatoes, and 18 g of agar in 1 L of natural seawater). Strain DT001 was further isolated from the collection plate and successively re-plated until a pure strain was obtained. The taxonomic identification of strain DT001 was carried out by the sequencing of the 1462 bp of 16S rRNA gene and compared to the nucleotide database of the National Center for Biotechnology Information (NCBI) using the Basic Local Alignment Search Tool (BLAST^®^) and showed 99% similarity with *Bacillus* sp. The sequence of strain DT001 was deposited in GenBank under accession number MG018253. A reference specimen of the octocoral (GLCO-100312-21) and bacterial strain *Bacillus* sp. DT001 are deposited at the INDICASAT Center for Biodiversity and Drug Discovery (CBDD).

### 4.3. Fermentation, Extraction, and Isolation of Surfactins

*Bacillus* sp. DT001 was inoculated in ten Erlenmeyer flasks containing 1 L of liquid medium M1. Erlenmeyer flasks were placed in an orbital shaker at 150 rpm at room temperature. After ten days, the culture broth was extracted with ethyl acetate (4 × 500 mL), and then, the organic layer was washed with distilled water (4 × 500 mL) to remove traces of the culture media. The organic extract was dried under reduced pressure to yield 281 mg of crude extract.

The extract (281 mg) was fractionated using C-18 solid-phase extraction cartridges eluting in a stepwise gradient of 20%, 40%, 60%, 80%, and 100% MeOH in water to yield five fractions (A–E). Fraction E (134.1 mg) was subjected to reverse-phase HPLC purification (Agilent Zorbax^®^ SB-C18 4.6 mm × 150.0 mm column, gradient from 80 to 95% MeOH/H_2_O for 50 min, then 95% MeOH/H_2_O for five minutes, and returning to the initial gradient 80% MeOH/H_2_O for ten minutes at 1.0 mL/min) to yield pumilacidin A (**1**) (36.2 mg, t_R_ 43.8 min) and pumilacidin C (**2**) (15.3 mg, t_R_ 50.6 min).

**Pumilacidin A** (**1**): white solid; [α]_D_^25^—11.2 (MeOH, *c* 0.28); UV (MeOH) λ_max_ (log ε) 206 nm (6.99); ^1^H-NMR (CD_3_OD, 400 MHz) 5.20 (1H, br), 4.71 (1H, br), 4.51, (1H, br), 4.48, (1H, br), 4.47, (1H, br), 4.37 (1H, br), 4.35 (1H, br), 4.29 (1H, Br), 2.66 (1H, dd, *J* = 4.4, 14.3 Hz), 2.48 (1H, dd, *J* = 8.0, 14.6 Hz), 2.10 (1H, br), 1.99, (1H, br), 1.96 (1H, br), 1.78, (1H), 1.68 (1H, br), 1.66 (8H, m), 1.55 (1H, br), 1.36 (4H, br), 1.34 (1H, br), 1.32 (14H, br), 1.25 (2H, br), 1.22 (2H, br), 0.99 (9H, br), 0.96 (9H, br), 0.95 (6H, br), 0.94 (6H, br), 0.91 (3H, br), 0.90 (3H, br); ^13^C-NMR (CD_3_OD, 100 MHz) 176.4 (C=O), 176.3 (C=O), 175.9 (C=O), 175.8 (C=O), 175.6 (C=O), 174.6 (C=O), 174.5 (C=O), 173.8 (C=O), 173.6 (C=O), 173.3 (C=O), 74.8 (CH–O), 59.2 (CH–N), 55.1 (CH–N), 55.0 (CH–N), 54.5 (CH–N), 54.4 (CH–N), 53.9 (CH–N), 52.7 (CH–NH), 43.1 (CH_2_), 42.5 (CH_2_), 42.2 (CH_2_), 42.0 (CH_2_), 41.8 (CH_2_), 41.1 (CH_2_), 39.2 (CH), 38.6 (CH_2_), 36.5 (CH), 36.2 (CH_2_), 31.9 (CH_2_), 31.8 (CH_2_), 31.6 (CH_2_), 31.4 (CH_2_), 31.4 (CH_2_), 31.2 (CH_2_), 30.0 (CH), 29.4 (CH_2_), 29.0 (CH_2_), 27.2 (CH_2_), 27.0 (CH_2_), 26.8 (CH), 26.8 (CH), 26.6 (CH), 24.4 (CH_3_), 24.2 (CH_3_), 24.1 (CH_3_), 23.9 (CH_3_), 23.4 (CH_3_), 23.2 (CH_3_), 22.9 (CH_3_), 22.5 (CH_3_), 20.5 (CH_3_), 17.0 (CH_3_), 12.7 (CH_3_), 12.6 (CH_3_); HRESITOF-MS *m/z* 1050.7030 [M + H]^+^ (calculated for C_54_H_96_N_7_O_13_).

**Pumilacidin C** (**2**): white solid; [α]_D_^25^—13.1 (MeOH, *c* 0.7); UV (MeOH) λ_max_ (log ε) 203 nm (7.05); ^1^H-NMR (CD_3_OD, 400 MHz) 5.21 (1H, br), 4.70 (1H, br), 4.51 (1H, br), 4.47 (1H, br), 4.38 (1H, br), 4.37 (1H, m), 4.36 (1H, br), 4.28 (1H, br), 2.65 (1H, dd, *J* = 3.7, 12.9 Hz), 2.47 (1H, dd, *J* = 8.1, 14.3 Hz), 2.10 (1H, m), 1.98 (1H, m), 1.97 (1H, m), 1.78 (1H, br), 1.68 (1H, br), 1.66 (8H, m), 1.55 (1H, br), 1.36 (4 H, br m), 1.34 (1H, br), 1.32 (18H, br m), 1.25 (2H, br), 1.22 (2H, br), 1.00 (9H, br), 0.97 (9H, br), 0.95 (3H, br), 0.94 (9H, br), 0.92 (3H, br), 0.90 (3H, br); ^13^C-NMR (CD_3_OD, 100 MHz) 176.3 (C=O), 175.8 (C=O), 175.6 (C=O), 174.6 (C=O), 174.6 (C=O), 174.6 (C=O), 173.8 (C=O), 173.6 (C=O), 173.3 (C=O), 173.3 (C=O), 74.8 (CH–O), 59.2 (CH–N), 55.1 (CH–N), 55.1 (CH–N), 54.6 (CH–N), 54.5 (CH–N), 53.9 (CH–N), 52.8 (CH–N), 43.1 (CH_2_), 42.5 (CH_2_), 42.3 (CH_2_), 42.0 (CH_2_), 41.8 (CH_2_), 41.1 (CH_2_), 39.2 (CH), 38.6 (CH_2_), 36.5 (CH), 36.2 (CH_2_), 31.9 (CH_2_), 31.9 (CH_2_), 31.6 (CH_2_), 31.6 (CH_2_), 31.6 (CH_2_), 31.4 (CH_2_), 31.4 (CH_2_), 31.2 (CH_2_), 30.0 (CH), 29.4 (CH_2_), 29.0 (CH_2_), 27.2 (CH_2_), 27.0 (CH_2_), 26.9 (CH), 26.8 (CH), 26.7 (CH), 24.4 (CH_3_), 24.2 (CH_3_), 24.1 (CH_3_), 23.9 (CH_3_), 23.4 (CH_3_), 23.2 (CH_3_), 23.0 (CH_3_), 22.5 (CH_3_), 20.5 CH_3_), 17.0 (CH_3_), 12.7 (CH_3_), 12.6 (CH_3_); HRESITOF-MS *m/z* 1078.7390 [M + H]^+^ (calculated for C_56_H_100_N_7_O_13_).

### 4.4. Tandem Mass Spectrometry Experiments

For the IT-FTICRMS analysis, the bacterial extracts were diluted 1:10 in the electrospray mixture (49.5% MeOH, 49.5% H_2_O, and 1% formic acid) and directly infused into the mass spectrometer as previously described [52]. For ESI-Q-TOF-MS analysis, extracts were diluted in methanol and analyzed using a Kinetex^®^ 1.7 μm, C_18_, 100 Å LC column 50 × 2.1 mm (Phenomenex^®^, Torrance, CA, USA). The gradient for the chromatographic analysis was carried out using a step (UPLC) run of methanol in acidified water (99.9% water and 0.1% formic acid), 7-min gradient from 90:10% MeOH/H_2_O to 100% MeOH, then 100% MeOH for 9 min, and returning to the initial condition 90:10% MeOH/H_2_O for 1 min at a flow rate of 0.3 mL/min throughout the run. MS spectra were acquired in positive ion mode in the range of 50–2250 *m/z* using data-dependent MS/MS fragmentation previously described [53]. Prior to collecting the data, an external calibration was performed using an Agilent ESI-L Low-Calibration Tuning Mix, and, throughout the runs, we used either hexakis (1H, 1H, 2H-difluoroethoxy), phosphazene (*m/z* 622.0290 [M + H]^+^) (Synquest Laboratories, Alachua, FL, USA), or reserpine (*m/z* 609.2807 [M + H]^+^) (Sigma-Aldrich, St. Louis, MO, USA) for lock mass calibration. 

### 4.5. Parasites and Cultures

The malaria parasite strain HB3 of *P. falciparum* was cultured following the method described by Haynes et al. [54] with some modifications. In brief, O+ erythrocytes were used in a complete medium that consisted of Roswell Park Memorial Institute medium (RPMI-1640) supplemented with 25 mM 4-(2-hydroxyethyl)-1-piperazineethanesulfonic acid (HEPES), 0.2% sodium bicarbonate, and 10% human serum. Blood was obtained from donors following the protocol approved by the Gorgas Memorial Institute bioethics committee. Cultures were maintained at 37 °C in a gas mixture of 5% CO_2_, 5% O_2_, and 90% N_2_ at 2% hematocrit, and synchronized with 0.3 M alanine and thermal cycling as described by Almanza et al. [55].

### 4.6. Cell Viability Assays

Vero epithelial cells (African monkey kidney epithelial cells) were seeded in 96-well plates using RPMI-1640 medium (Sigma-Aldrich, USA) supplemented with 10% fetal bovine serum (FBS; Gibco, Invitrogen, Carlsbad, CA, USA) and 1% penicillin/streptomycin (stock mix at 10,000 units penicillin and 10 mg streptomycin per mL in 0.9% NaCl). The assays were based on those described by Mosmann et al. [56]. Briefly, cells were allowed to grow for 24 h before adding pumilacidin samples. A negative control without any sample, but with an equal volume of the vehicle, was placed in all plates and counted as 100% growth. All samples were incubated for three days before staining and examining for the reduction of 3-(4,5-dimethylthiazol-2-yl)-2,5-diphenyltetrazolium bromide (MTT) after incubating it with the cells for a period of four hours. The ELISA plate reader used to measure color was used at a wavelength of 570 nm. The IC_50_ was calculated using the Data Analysis Wizard complement of Excel 2000 (Microsoft, Seattle, WA, USA).

### 4.7. IC_50_ Calculation for Pumilacidins A and C

The IC_50_ of the compounds was calculated using four concentrations of surfactin samples (10, 2, 0.4, and 0.08 μM) using chloroquine as a positive control (CQ) (1000, 100, and 0.1 nM) and parasites at two different stages (ring stage 6–14 h, and schizont stage 38–48 h post invasion). Parasite cultures were placed in 96-well plates and pumilacidins were added and incubated for 48 h. After this period, a mix of the PicoGreen DNA fluorescent dye (Invitrogen, Carlsbad, CA, USA) was added to a final concentration of 1%, and after 30 min incubation, the signal was monitored on a fluorescence plate reader as described in Corbett et al. [57] The IC_50_ was calculated using the Data Analysis Wizard complement of Excel 2000 (Microsoft, Seattle, WA, USA).

### 4.8. Anti Plasmodium Assays

Following treatment with 10 μM of pumilacidins, schizont-synchronized cultures (between 38–48 h post invasion) with 2% parasitemia were seeded in 96-well plates for 24 h. For parasite morphology analysis, Giemsa-stained thin smears were prepared from each well and the number of infected erythrocytes in at least 1000 erythrocytes was determined by light microscopy after blinding the slides to the microscopist.

To determine viability, we analyzed the morphology of the parasites under a microscope and measured the parasitemia using flow cytometry, staining with 2 µg/mL Hoechst 33342 (Invitrogen, Carlsbad, CA, USA) prior to fixation with 4% paraformaldehyde. The flow cytometry analysis was carried out by exciting the samples with a UV laser. The data were analyzed with FloMax^®^ software version 2.7 (Quantum Analysis GbmH, Münster, Germany). A target gate was selected after analyzing uninfected erythrocytes. The fluorescence-positive events in that gate were taken as infected parasites.

### 4.9. Hemolysis Assay

The test was performed in 96-well plates following the method described by Costa-Lotufo et al. [58] with some modifications. The negative control contained only red blood cells in RPMI medium. The compounds were tested at the highest concentration used in this work (10 µM). As a positive control, 20 µL of 0.1% Triton X-100 (in RPMI) was used to obtain 100% hemolysis. Compounds were incubated for 24 h at 37 °C and centrifuged. The supernatant was removed, and the released hemoglobin was measured spectroscopically for its absorbance at 415 nm.

### 4.10. Propidium Iodide (PI) Incorporation Assay

The protocol followed was as per the manufacturer’s instructions (BD Pharmingen^TM^). In brief, 200 µL of parasite culture at 2% parasitemia and 2% hematocrit, or 200 µL of red blood cells at 2% hematocrit were incubated with pumilacidin compounds for 3 h at 37 °C. Untreated samples were kept in the same plate. After incubation, 2 µL of PI was added with gentle homogenization and incubated for 15 min at room temperature under dark conditions. Fluorescence was read in a plate-ready fluorometer at excitation and emission wavelengths of 485/20 and 645/40 nm, respectively.

### 4.11. Mitochondrial Membrane Potential (∆ψ) Measurements in P. falciparum

Changes in membrane potential were measured by staining *P. falciparum* cultures with the DiOC6(3) dye at 10 nM (Thermo Fisher, Waltham, MA, USA) using flow cytometry. Samples were incubated at 37 °C for 45 min in the dark. Following incubation, cells were washed and suspended in 200 μL of phosphate-buffered saline (PBS) and analyzed by flow cytometry immediately after. The mitochondrial membrane disrupter, CCCP (50 µM final concentration), incubated for 1 h, was used as a positive control. The green fluorescence intensity of DiOC6-stained cells was calculated using FCS Express 4 (De Novo Software, Glendale, CA, USA). As a control, uninfected red blood cells were used in all treatments applied and their signal subtracted from the signal of the infected red blood cells [59].

### 4.12. Intracellular Reactive Oxygen Species (ROS) Production in P. falciparum

Intracellular ROS formation was measured by flow cytometry with chloromethyl dichlorodihydrofluorescein diacetate (CM-H_2_DCFDA; Molecular Probes^®^) reactive dye. Hydrogen peroxide, a potent oxygen radical inducer, was used as a positive control at a final concentration of 100 μM. The level of ROS present was inferred through the amount of oxidized DCF [60]. As a control, uninfected red blood cells were used in all treatments applied. *P*. *falciparum* cultures at 2% parasitemia were incubated for 30 min at room temperature in the dark with PBS containing 10 μM CM-H_2_DCFDA. Cultures were washed once with PBS, resuspended in RPMI-1640, and allowed to recover for 30 min in a 37 °C incubator. After incubation, dye-preloaded cultures were incubated with pumilacidins A and C at 10 μM for 3, 6 and 9 h. The intracellular ROS production was measured by flow cytometry with a 488-nm argon laser as a light source. Experiments were repeated at least two times, in duplicate, and the results presented are representative of these experiments.

### 4.13. Calcium Measurements

FURA 2AM, a membrane-permeable calcium indicator fluorescent probe, was used to study changes in parasite cytosolic calcium after treatment with pumilacidins. Parasites were isolated from RBCs using 0.05% saponin, and were stained with 10 µM Fura 2 AM in ringer’s solution and 0.01% pluronic acid for 2 h at 37 °C in the dark. After the 2-h incubation, cells were centrifuged at 2000× *g* for 1 min, washed with PBS once, and resuspended in 175 µL of RPMI and 25 µL of pumilacidin samples in a final concentration of 10 µM. After this, fluorescence was measured in the fluorometer. The calcium levels, obtained by measuring cell fluorescence intensity at excitation and emission wavelengths of 360 and 510 nm, respectively, were determined at 10, 30, 60, and 90 min after introduction of surfactins.

### 4.14. Transduction Pathways

Three different inhibitors of growth signal transduction through inhibition of the IP3 signaling pathway were used: 3MA 0.2 μM (SIGMA); LY29400 5 μM (SIGMA); bisindolylmaleimide I (PKC inhibitor) 4 μg/mL (Abcam, Cambridge, MA, USA). All inhibitors were diluted in culture media. Parasites were pre-incubated with inhibitors for 3 h, then washed and resuspended in RPMI media supplemented with 10% serum before being exposed to standard treatment with 10 μM pumilacidin. Control samples containing the inhibitors, but not pumilacidins, were used. After 24 h of incubation at 37 °C, the parasitemia was measured by flow cytometry with Hoechst H33342 staining at 2 μg/mL, and their morphology was analyzed by Giemsa smears.

## Figures and Tables

**Figure 1:**
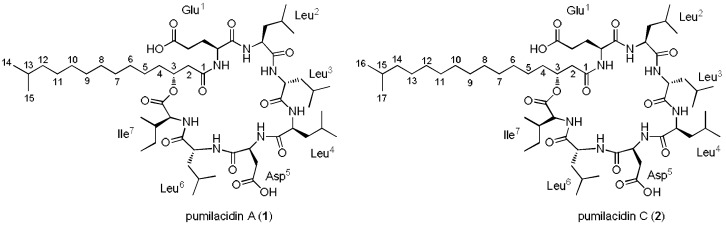
Structures of pumilacidins **A** and **C**.

**Table 1:** Antiplasmodial activity of pumilacidins on different parasite stages. The data shown are from three experiments run in duplicate. IC_50_—half maximal inhibitory concentration; SEM—standard error of the mean.

Compound	Parasite Stage	IC_50_ (μM)	SEM
Pumilacidin A	schizont	8.34	0.97
	ring	15.44	3.9
Pumilacidin C	schizont	7.75	1.74
	ring	19.59	4.4

**Table 2:** The cytotoxic activity of pumilacidins on African green monkey kidney epithelial cells (Vero cells). Data are the means of one experiment run in duplicate.

Compound	IC_50_ (μM)	SEM
Pumilacidin A	28.42	9.86
Pumilacidin C	26.07	10.10

**Table 3:** Inositol triphosphate (IP3)-related signal transduction pathway inhibitors.

Inhibitor	Nomenclature Used	Target
3-Methyladenine (3MA)	Inhibitor 1	Phosphatidylinositol 3-kinases (PI3Ks) I and III
LY29400	Inhibitor 2	PI3-kinase I (PI3K)
Protein kinase C (PKC) inhibitor (bisindolylmaleimide I)	Inhibitor 3	PKC

## Supplementary Materials

The following are available online, Figures S1 to S18.
