# Supplementary material for: Pumilacidins from the Octocoral-Associated Bacillus sp. DT001 Display Anti-Proliferative Effects in Plasmodium falciparum
Source: Molecules. 2018 Aug 29;23(9):2179. doi: 10.3390/molecules23092179 (PMC6225264; doi:10.3390/molecules23092179)

Supplementary data

Pumilacidins from the octocoral-associated *Bacillus* sp. DT001 display anti proliferative effect in *P. falciparum* via mitochondrial dysfunction and possible survival signaling suppression.

Daniel Torres-Mendoza [1, 2], Lorena M. Coronado [3], Laura Pineda R.[3], Héctor M. Guzmán [4], Pieter C. Dorrestein[5, 6], Carmenza Spadafora [3,*] and Marcelino Gutiérrez [1,*]

[1] Centro de Biodiversidad y Descubrimiento de Drogas, Instituto de Investigaciones Científicas y Servicios de Alta Tecnología (INDICASAT AIP), Panamá, Apartado 0843-01103, República de Panamá.
[2] Department of Biotechnology, Acharya Nagarjuna University, Nagarjuna Nagar, Guntur 522510, India
[3] Centro de Biología Celular y Molecular de Enfermedades, INDICASAT AIP, Panamá, Apartado 0843-01103, República de Panamá.
[4] Smithsonian Tropical Research Institute, Balboa, Ancón, P.O. Box 0843-03092, República de Panamá.
[5] Collaborative Mass Spectrometry Innovation Center, Skaggs School of Pharmacy and Pharmaceutical Sciences, University of California at San Diego, California, 92093, United States.
[6] Department of Pharmacology, University of California at San Diego, California, 92093, United States.

Figure S1. Pumilacidin A, ^{1}H NMR spectrum

Figure S2. Pumilacidin A, ^{13}C NMR spectrum

Figure S3. Pumilacidin A, ^{13}C NMR-DEPT135 spectrum

Figure S4. Pumilacidin A, ^{13}C NMR-DEPT90 spectrum

Figure S5. Pumilacidin A, ^{1}H-^{1}H-COSY spectrum

Figure S6. Pumilacidin A, HSQC spectrum

Figure S7. Pumilacidin A, HMBC spectrum

Figure S8. Pumilacidin C, ^{1}H NMR spectrum

Figure S9. Pumilacidin C, ^{13}C NMR spectrum

Figure S10. Pumilacidin C, ^{13}C NMR-DEPT135 spectrum

Figure S11. Pumilacidin C, ^{13}C NMR-DEPT90 spectrum

Figure S12. Pumilacidin C, ^{1}H-^{1}H-COSY spectrum

Figure S13. Pumilacidin C, HSQC spectrum

Figure S14. Pumilacidin C, HMBC spectrum

Figure S15. Pumilacidin A, HRESITOF-MS

FigureS16. Pumilacidin A, MS/MS spectrum

Figure S17. Pumilacidin C, HRESITOF-MS

Figure S18. Pumilacidin C, MS/MS spectrum

Figure S1. Pumilacidin A, ^{1}H NMR spectrum

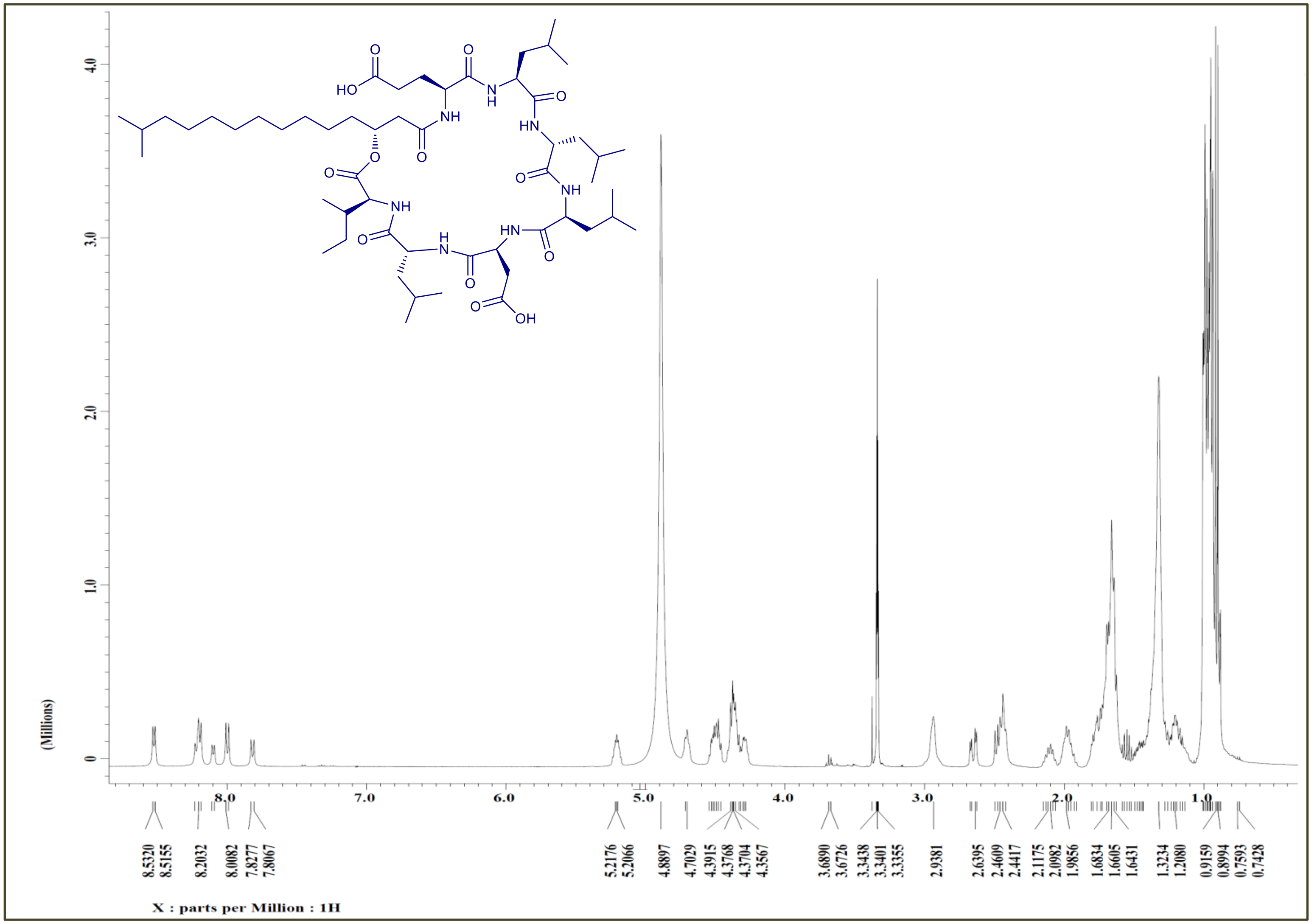

Figure S2. Pumilacidin A, ^{13}C NMR spectrum

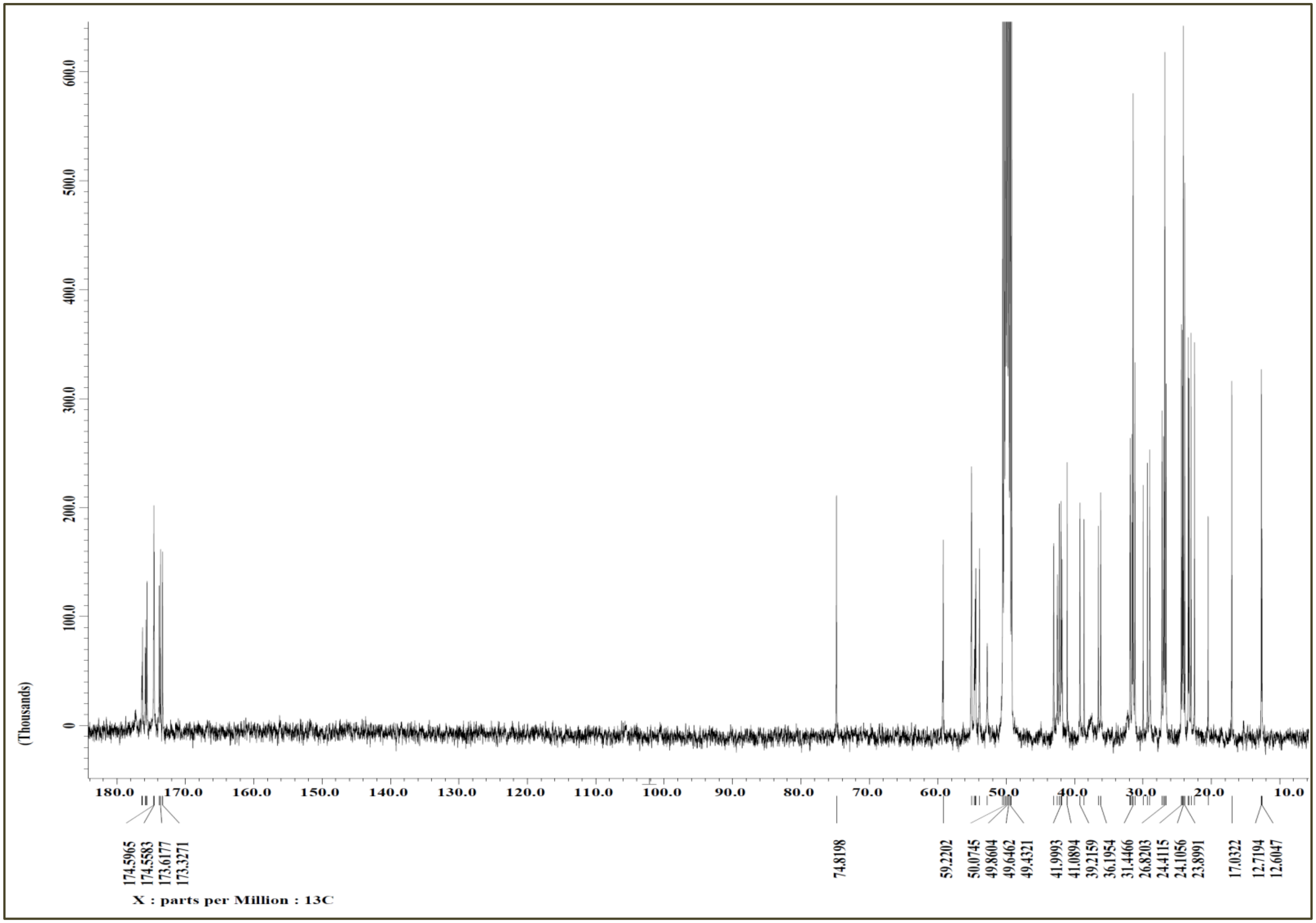

Figure S3. Pumilacidin A, ^{13}C NMR-DEPT135 spectrum

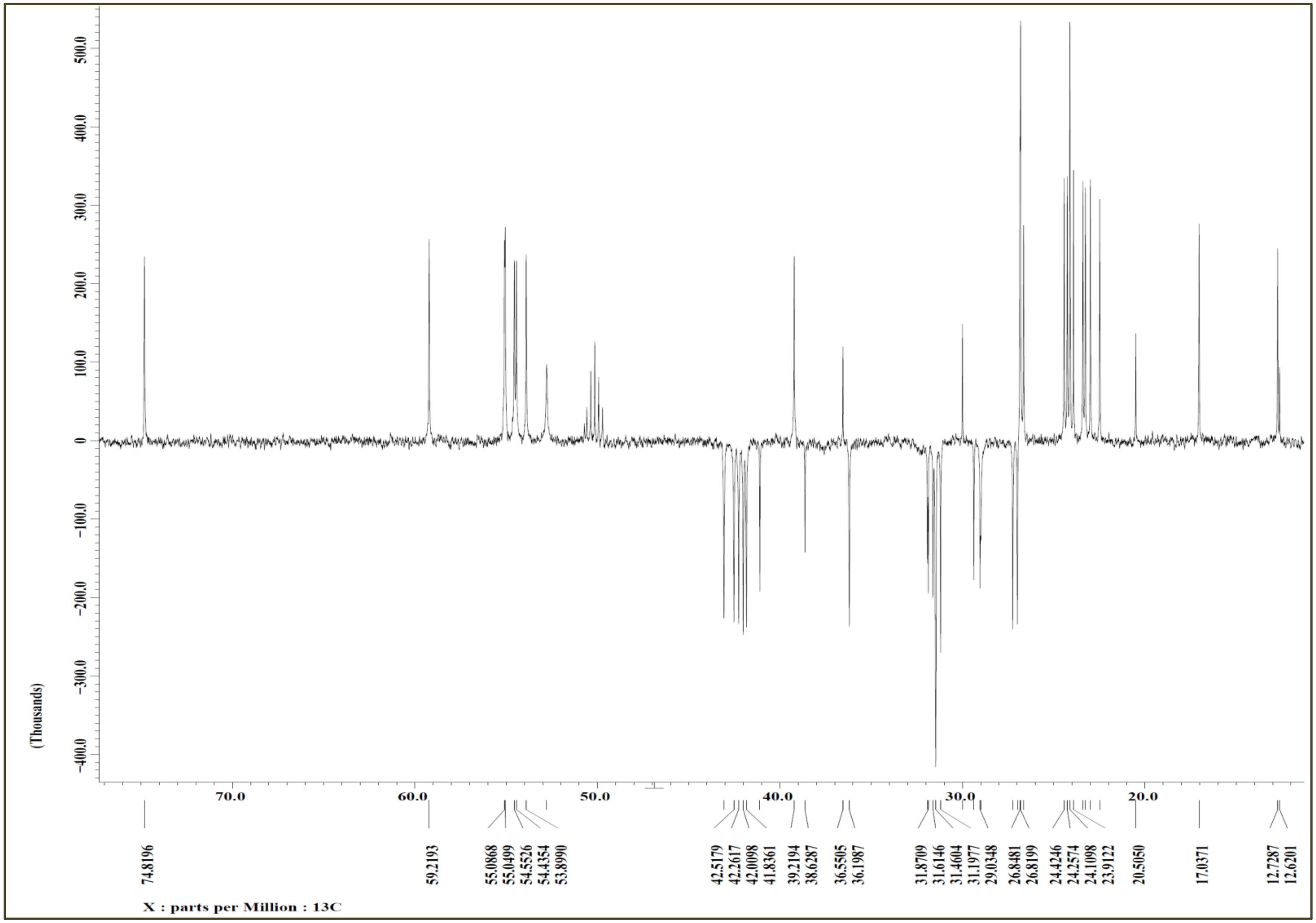

Figure S4. Pumilacidin A, ^{13}C NMR-DEPT90 spectrum

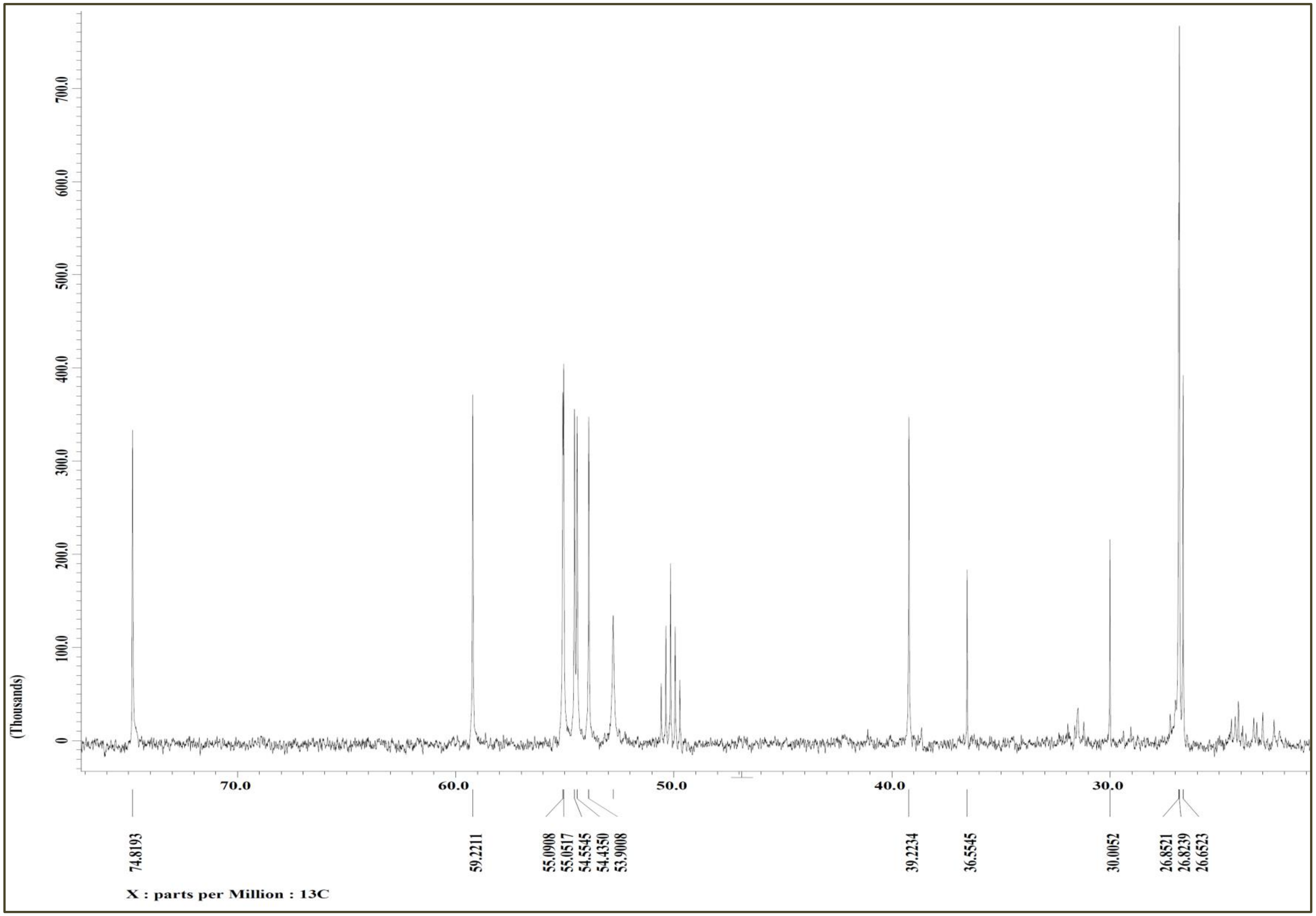

Figure S5. Pumilacidin A, ^{1}H-^{1}H-COSY spectrum

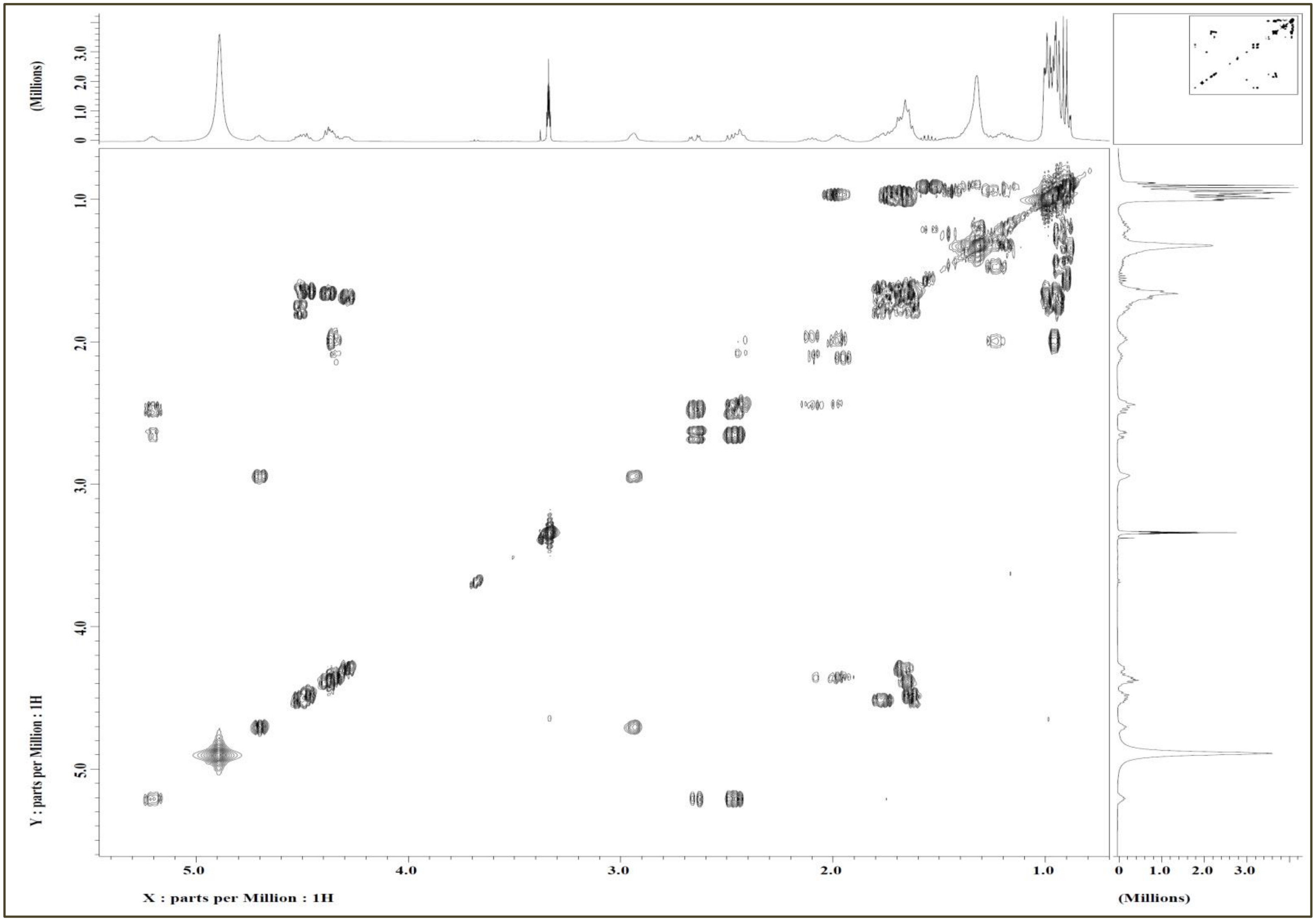

Figure S6. Pumilacidin A, HSQC spectrum

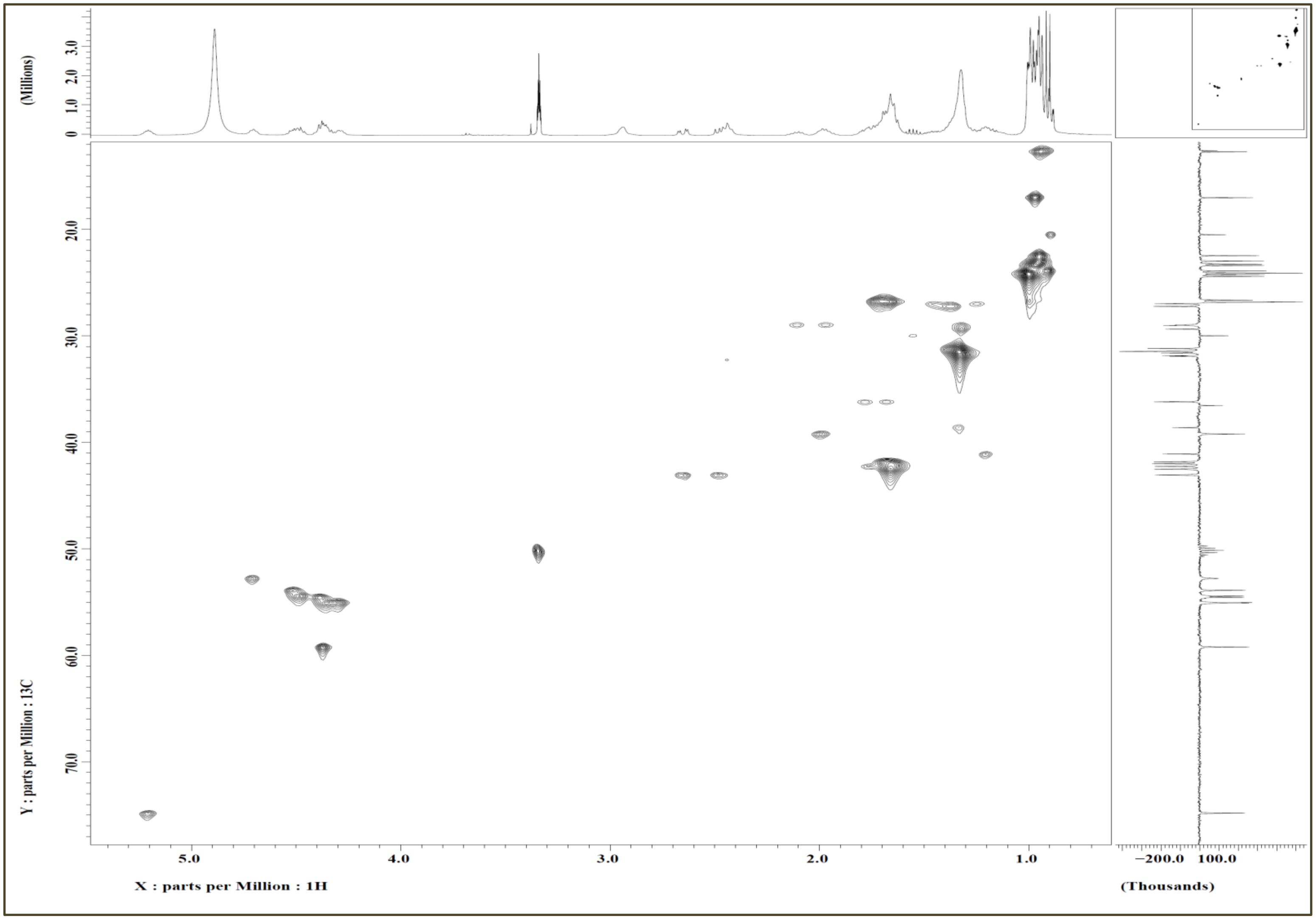

Figure S7. Pumilacidin A, HMBC spectrum

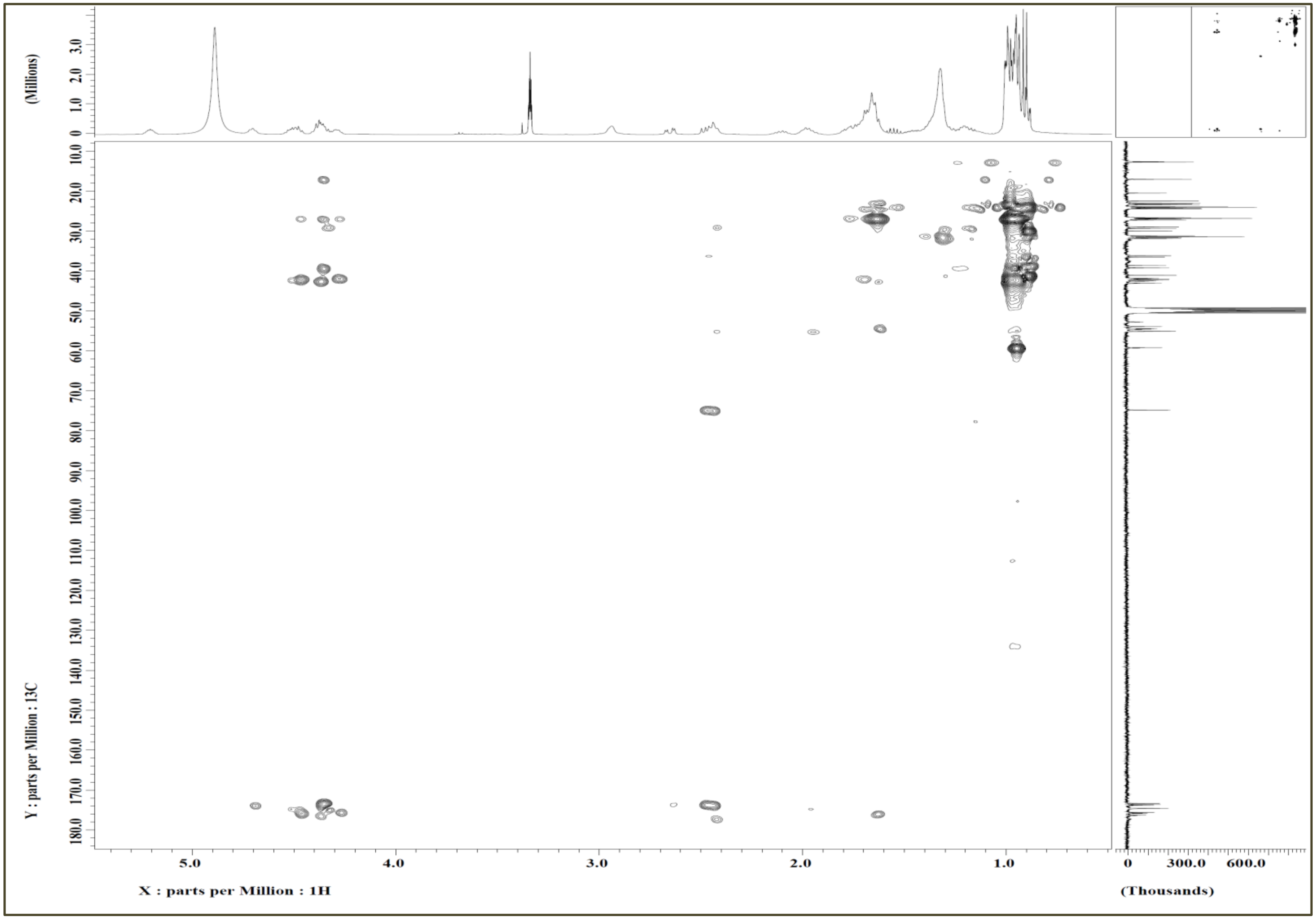

Figure S8. Pumilacidin C, ^{1}H NMR spectrum

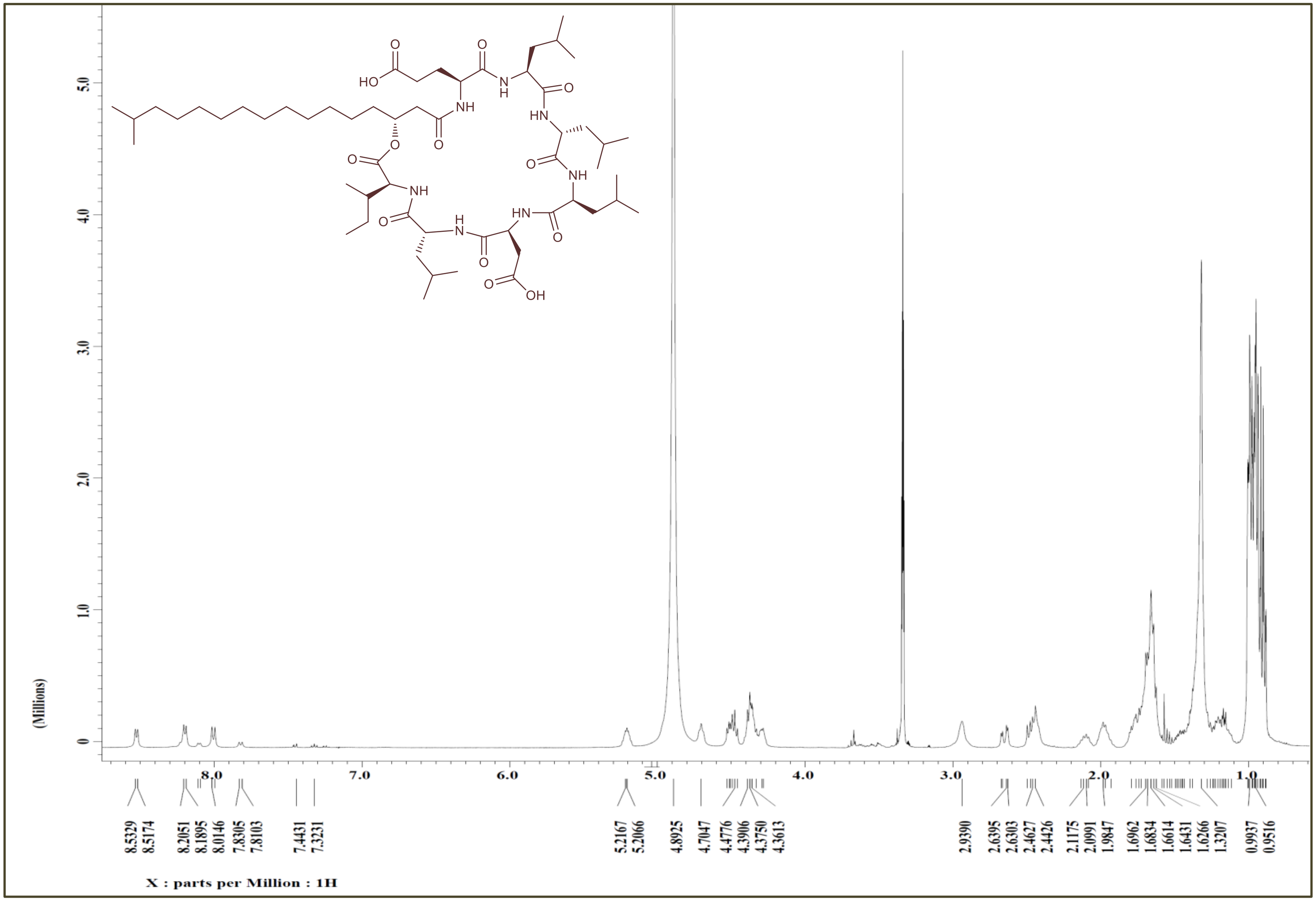

Figure S9. Pumilacidin C, ^{13}C NMR spectrum

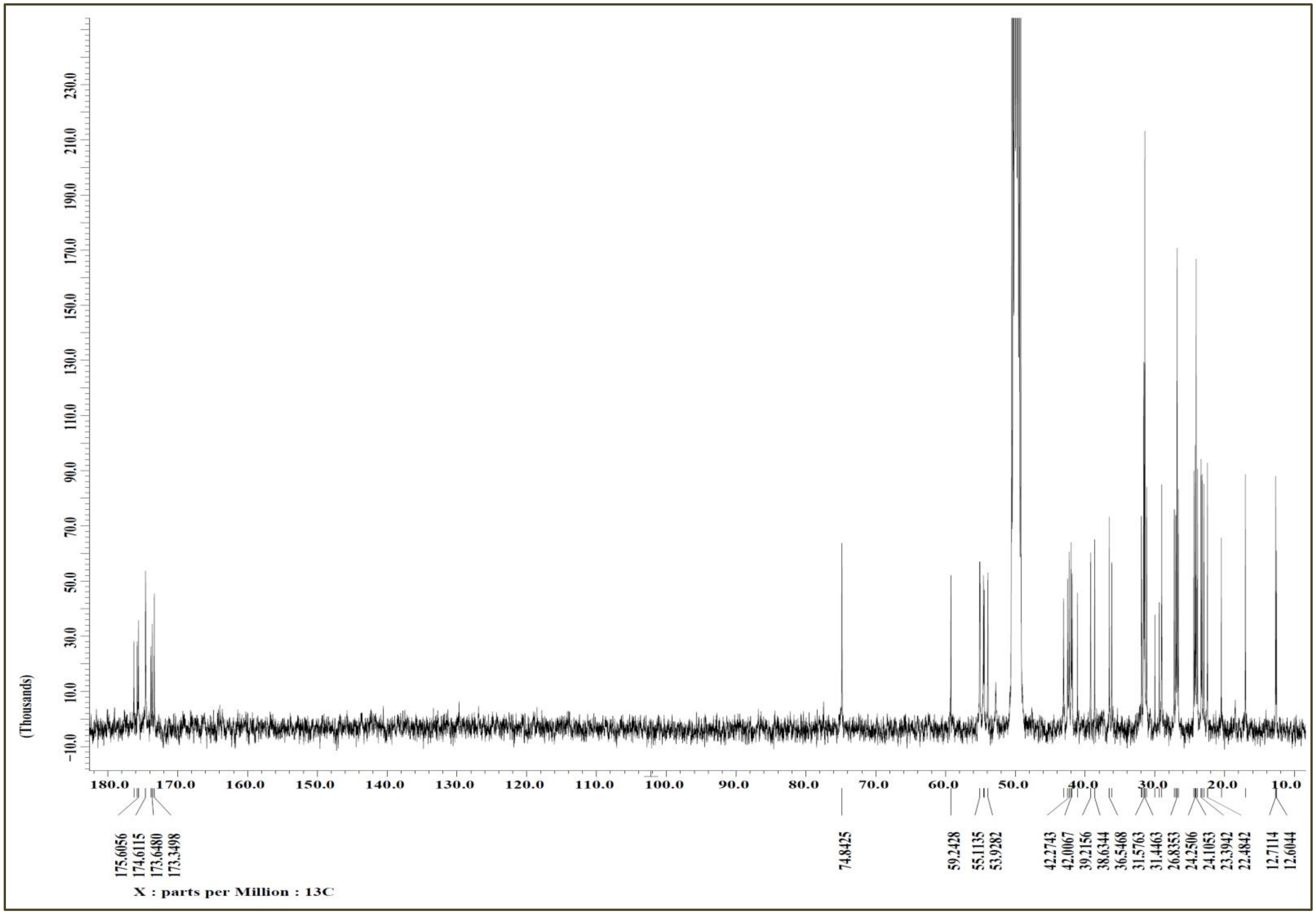

Figure S10. Pumilacidin C, ^{13}C NMR-DEPT135 spectrum

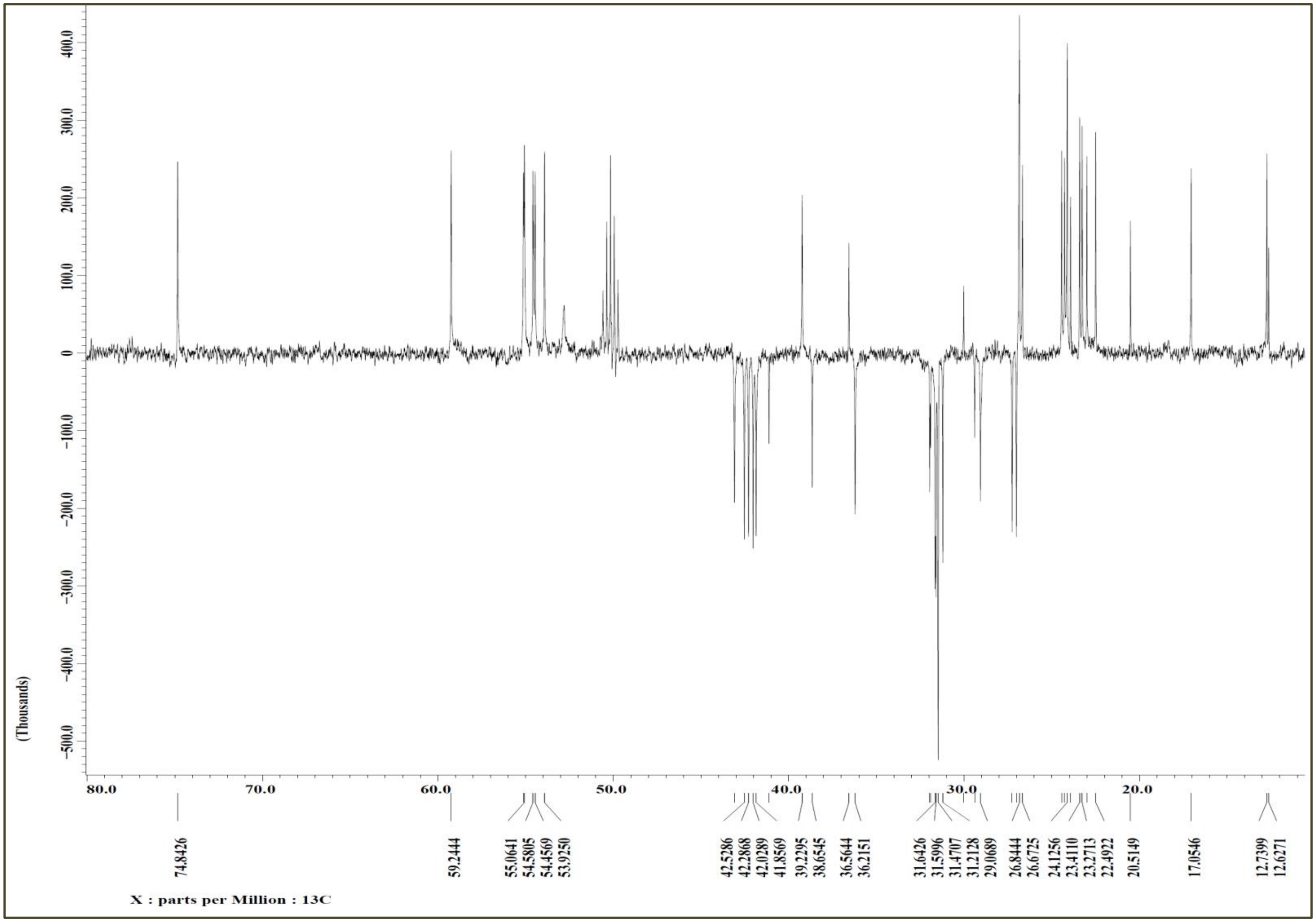

Figure S11. Pumilacidin C, ¹³C NMR-DEPT90 spectrum

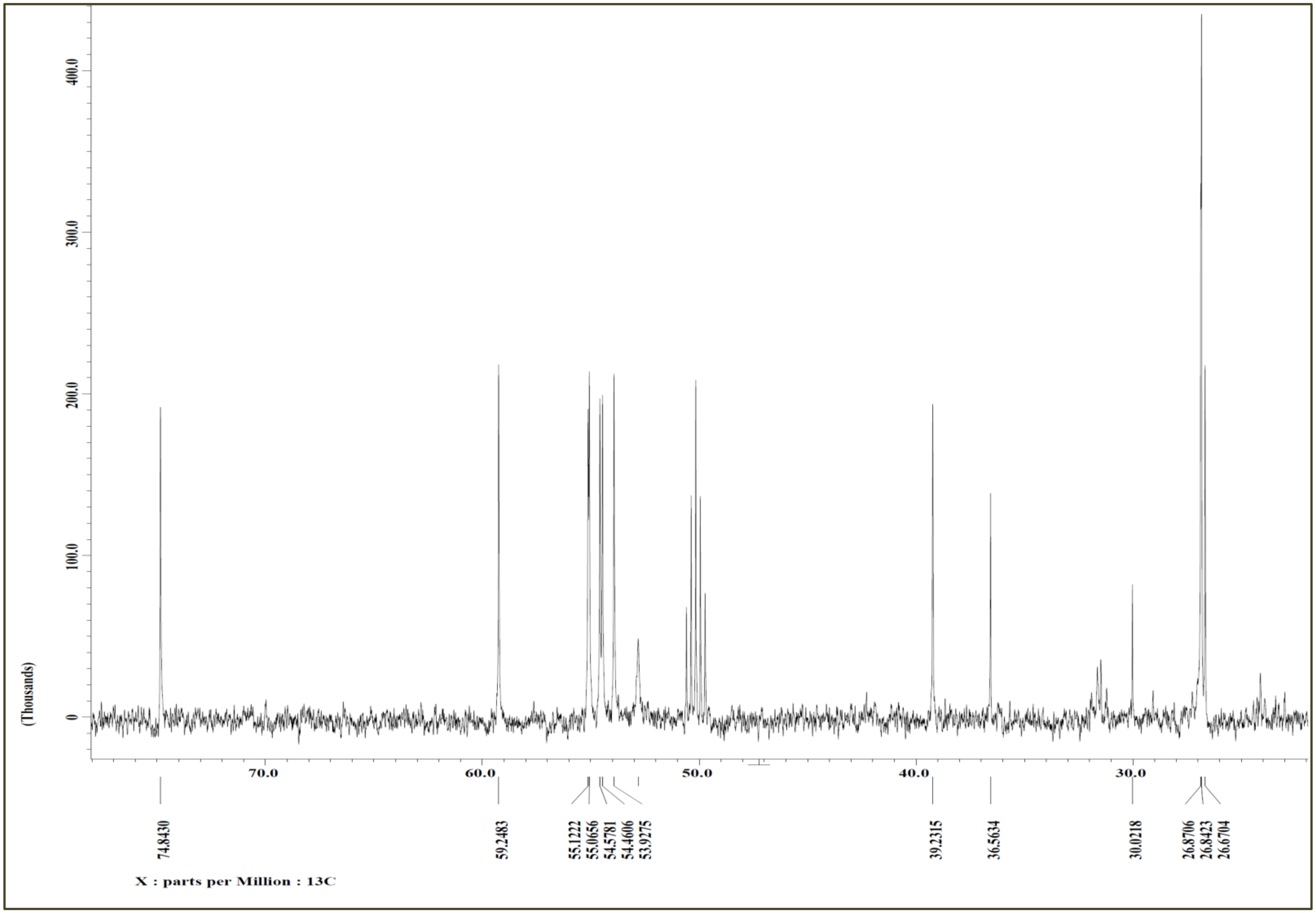

Figure S12. Pumilacidin C, ^{1}H-^{1}H-COSY spectrum

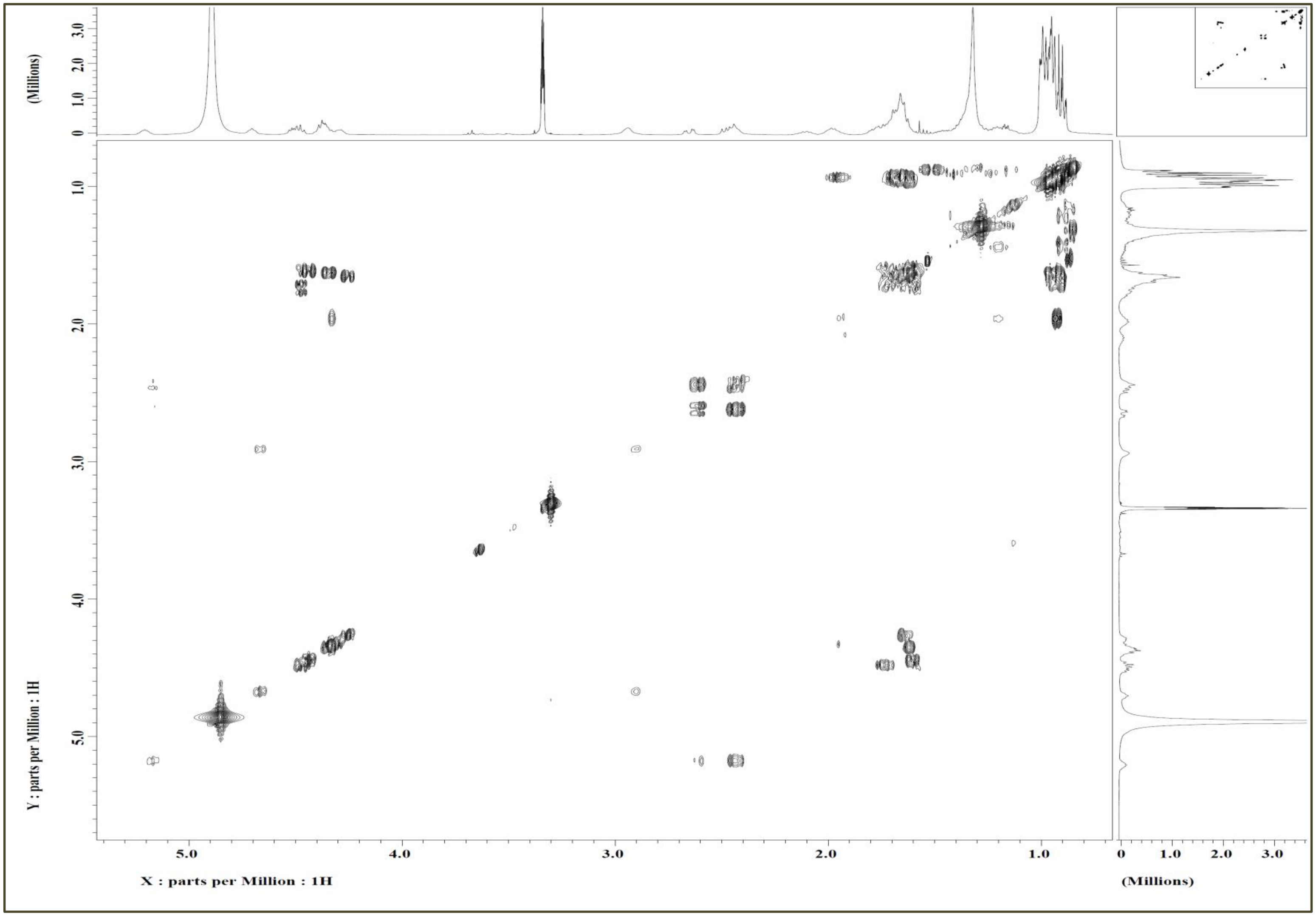

Figure S13. Pumilacidin C, HSQC spectrum

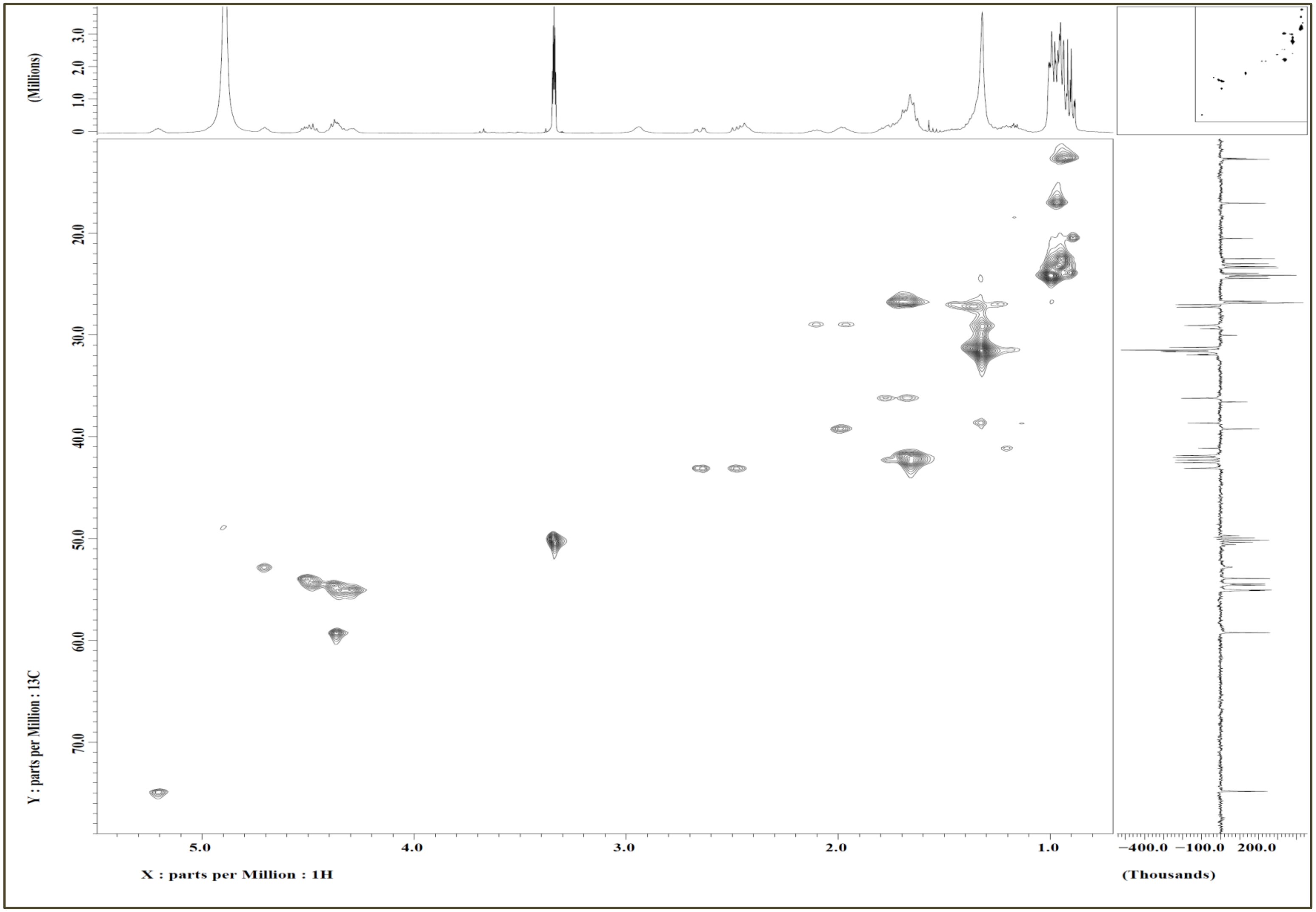

Figure S14. Pumilacidin C, HMBC spectrum

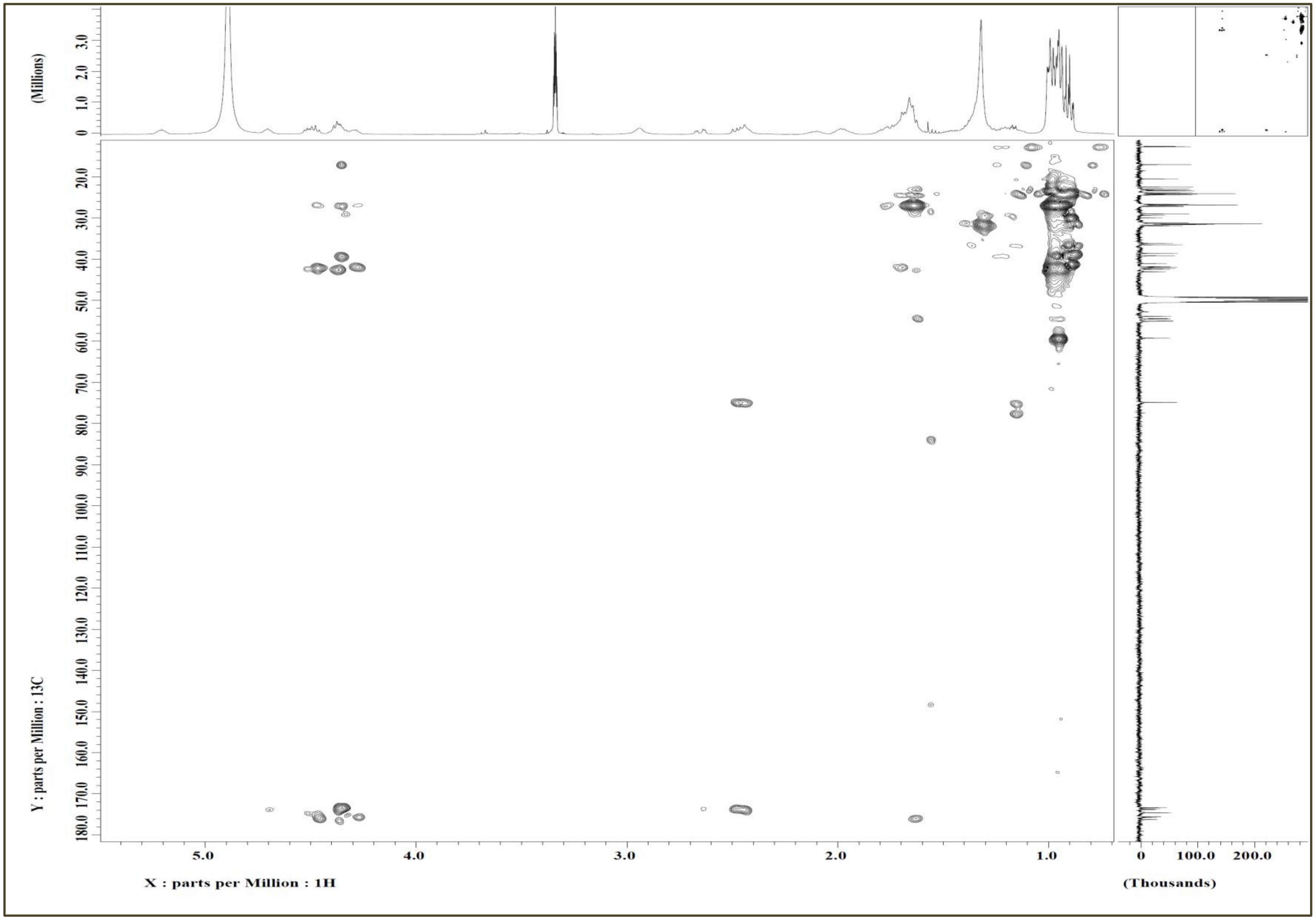

Figure S15. Pumilacidin A, HRESITOF-MS

Mass Spectrum SmartFormula Report

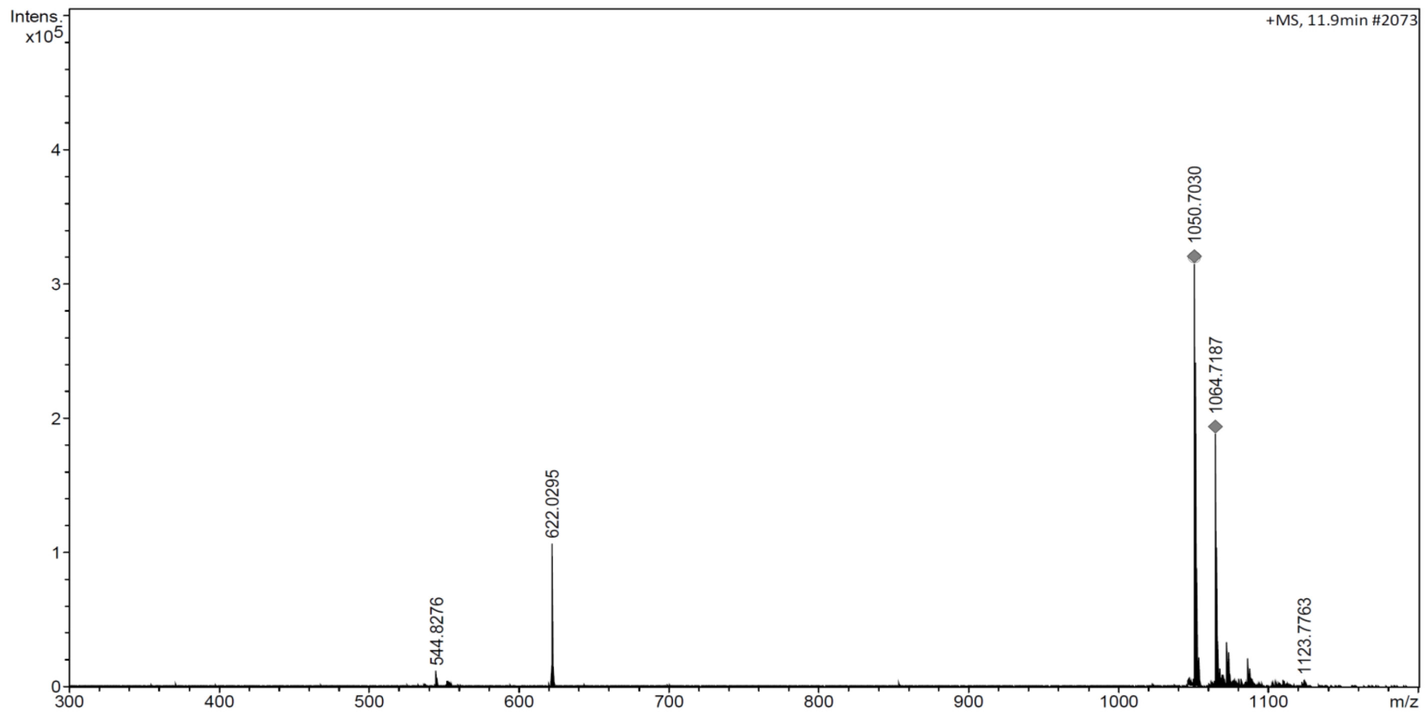

Meas. m/z	#	Ion Formula	m/z	err [ppm]	mSigma	# Sigma	Score	rdb	e⁻ Conf	N-Rule
1050.7030	1	$C_{54}H_{96}N_7O_{13}$	1050.7061	-3.0	69.3	1	100.00	10.5	even	ok

Figure S16. Pumilacidin A, MS/ MS spectrum

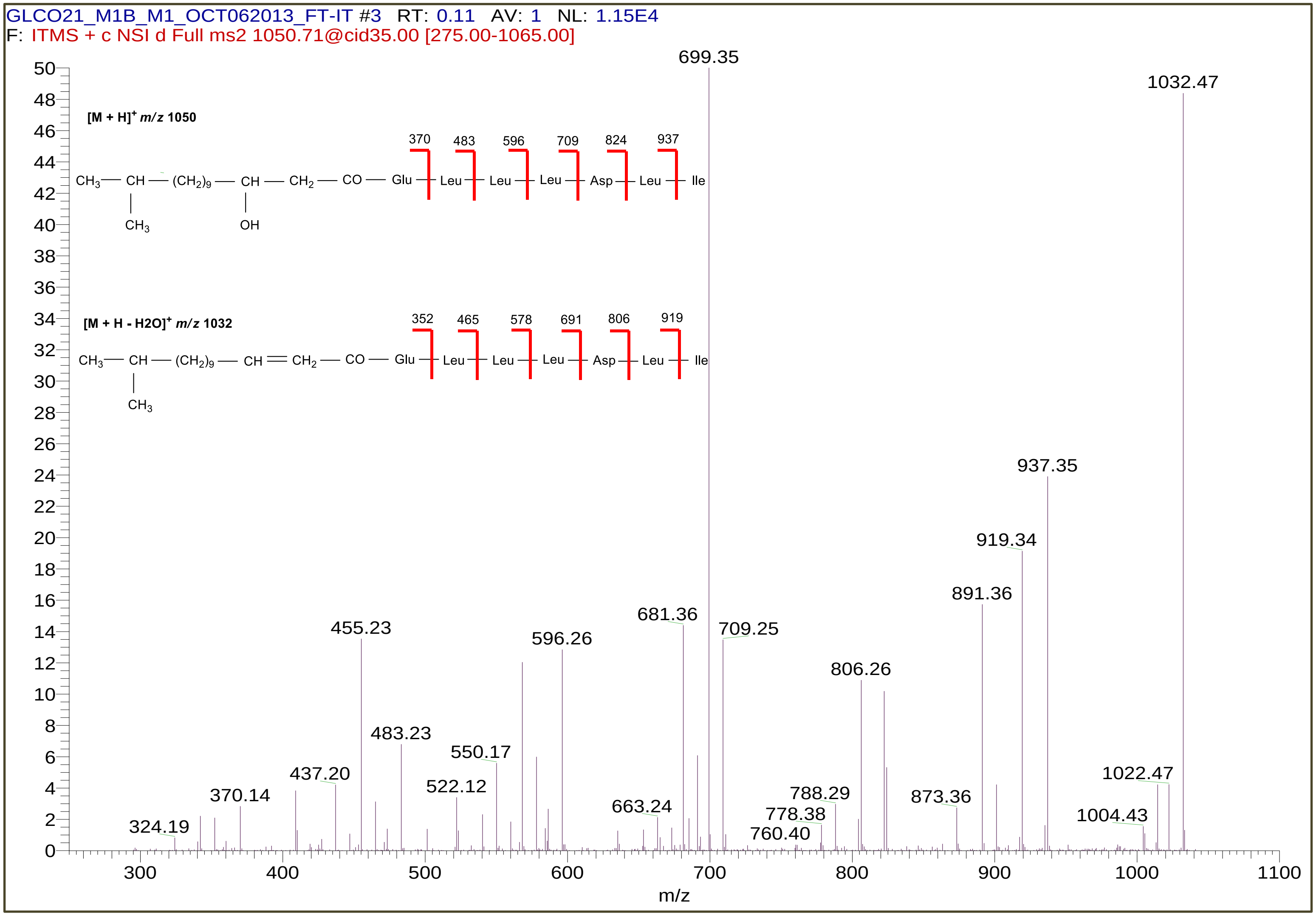

Figure S17. Pumilacidin C, HRESITOF-MS

Mass Spectrum SmartFormula Report

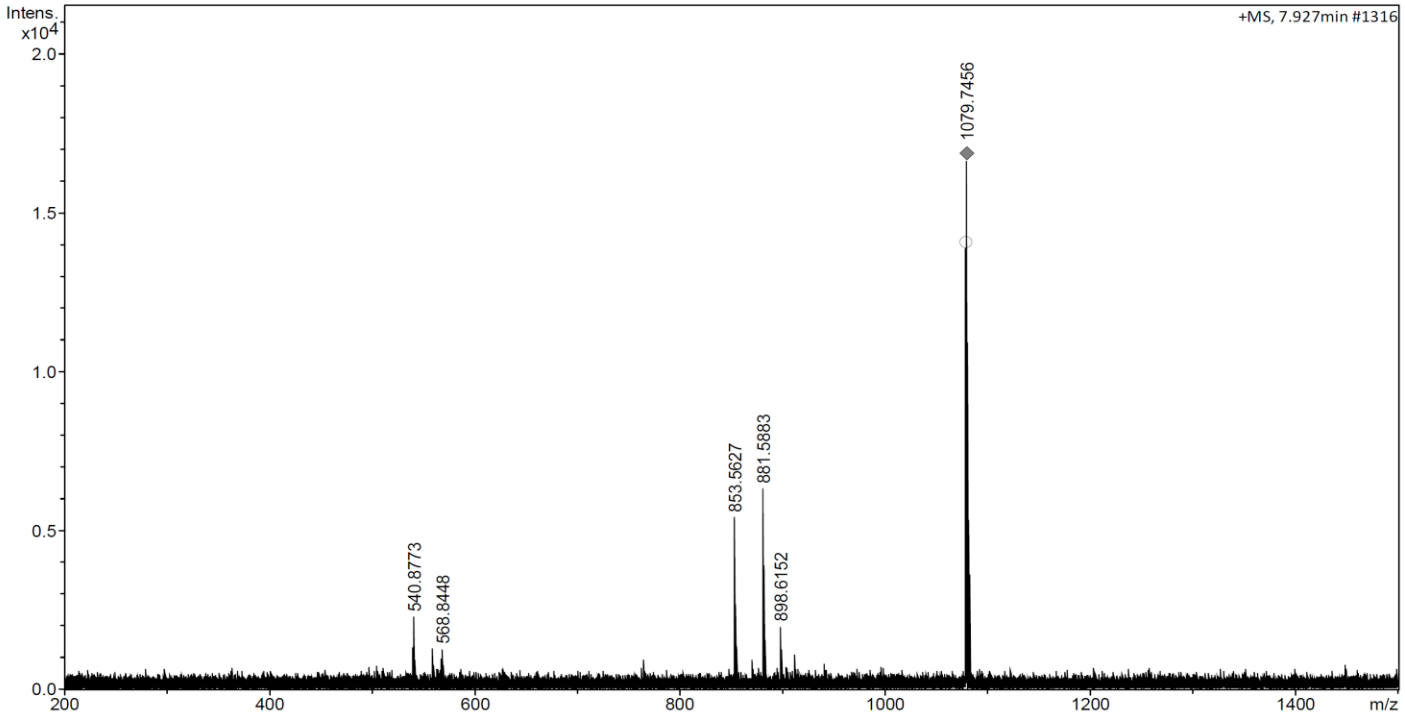

Meas. m/z	#	Ion Formula	m/z	err [ppm]	mSigma	# Sigma	Score	rdb	e⁻ Conf	N-Rule
1078.7390	1	C56H100N7O13	1078.7374	1.5	294.7	1	100.00	10.5	even	ok

Figure S18. Pumilacidin C, MS/ MS spectrum

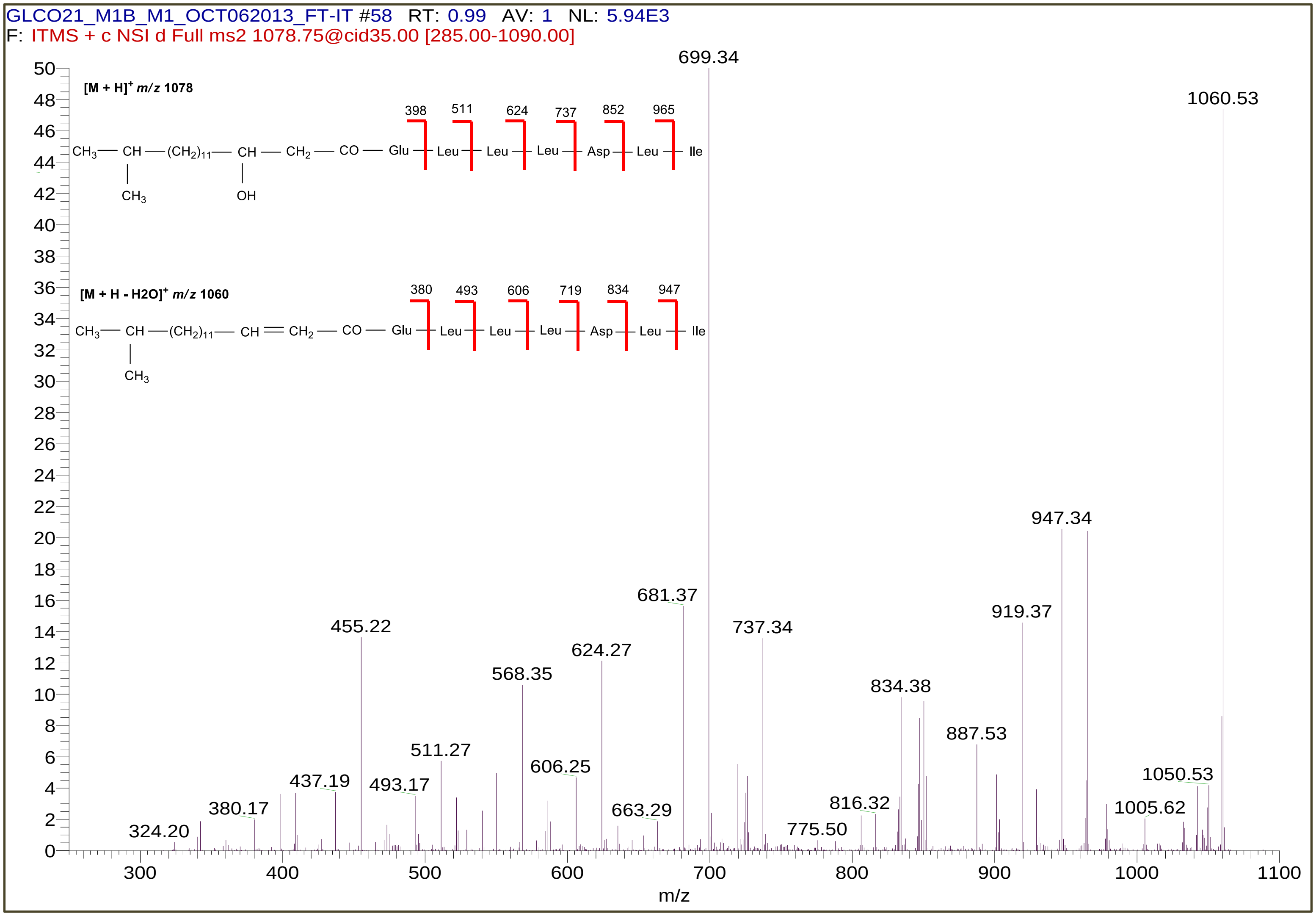

Supplement: Supplementary file 1 [file molecules-23-02179-s001.pdf]
